# Genomic and epigenomic evolution of acquired resistance to combination therapy in esophageal squamous cell carcinoma

**DOI:** 10.1172/jci.insight.150203

**Published:** 2021-09-08

**Authors:** Qingjie Min, Yan Wang, Qingnan Wu, Xianfeng Li, Huajing Teng, Jiawen Fan, Yiren Cao, Pingsheng Fan, Qimin Zhan

**Affiliations:** 1 Key Laboratory of Carcinogenesis and Translational Research (Ministry of Education/Beijing), Laboratory of Molecular Oncology, Peking University Cancer Hospital & Institute, Beijing, China.; 2 Department of Gastroenterology, Daping Hospital, Army Medical University (Third Military Medical University), Chongqing, China.; 3 Key Laboratory of Carcinogenesis and Translational Research (Ministry of Education/Beijing), Department of Radiation Oncology, Peking University Cancer Hospital and Institute, Beijing, China.; 4 Department of Medical Oncology, Anhui Provincial Cancer Hospital, Hefei, China.; 5 Institute of Cancer Research, Shenzhen Bay Laboratory, Shenzhen, China.; 6 Research Unit of Molecular Cancer Research, Chinese Academy of Medical Sciences, Beijing, China.

**Keywords:** Oncology, Clonal selection, Genetic variation, Molecular genetics

## Abstract

**BACKGROUND:**

Targeted arterial infusion of verapamil combined with chemotherapy (TVCC) is an effective clinical interventional therapy for esophageal squamous cell carcinoma (ESCC), but multidrug resistance (MDR) remains the major cause of relapse or poor prognosis, and the underlying molecular mechanisms of MDR, temporal intratumoral heterogeneity, and clonal evolutionary processes of resistance have not been determined.

**METHODS:**

To elucidate the roles of genetic and epigenetic alterations in the evolution of acquired resistance during therapies, we performed whole-exome sequencing on 16 serial specimens from 7 patients with ESCC at every cycle of therapeutic intervention from 3 groups, complete response, partial response, and progressive disease, and we performed whole-genome bisulfite sequencing for 3 of these 7 patients, 1 patient from each group.

**RESULTS:**

Patients with progressive disease exhibited a substantially higher genomic and epigenomic temporal heterogeneity. Subclonal expansions driven by the beneficial new mutations were observed during combined therapies, which explained the emergence of MDR. Notably, *SLC7A8* was identified as a potentially novel MDR gene, and functional assays demonstrated that mutant *SLC7A8* promoted the resistance phenotypes of ESCC cell lines. Promoter methylation dynamics during treatments revealed 8 drug resistance protein-coding genes characterized by hypomethylation in promoter regions. Intriguingly, promoter hypomethylation of *SLC8A3* and mutant *SLC7A8* were enriched in an identical pathway, protein digestion and absorption, indicating a potentially novel MDR mechanism during treatments.

**CONCLUSION:**

Our integrated multiomics investigations revealed the dynamics of temporal genetic and epigenetic inter- and intratumoral heterogeneity, clonal evolutionary processes, and epigenomic changes, providing potential MDR therapeutic targets in treatment-resistant patients with ESCC during combined therapies.

**FUNDING:**

National Natural Science Foundation of China, Science Foundation of Peking University Cancer Hospital, CAMS Innovation Fund for Medical Sciences, Major Program of Shenzhen Bay Laboratory, Guangdong Basic and Applied Basic Research Foundation, and the third round of public welfare development and reform pilot projects of Beijing Municipal Medical Research Institutes.

## Graphical abstract

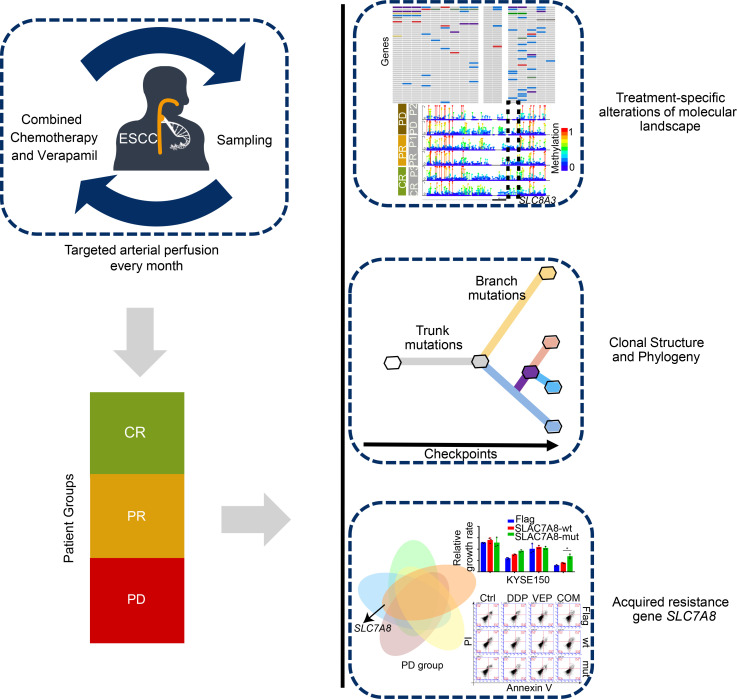

## Introduction

Esophageal cancer is the seventh most common cancer in terms of incidence and the sixth most fatal cancer worldwide. Eastern and southern Africa and eastern Asia rank as the top 3 regions based on age-standardized incidence rates according to the statistics from 185 countries (1). Although the overall 5-year survival rate has risen in recent years, it remains at a low level, ranging from 15% to 25% (2, 3). Esophageal squamous cell carcinoma (ESCC), the predominant histologic subtype of esophageal cancer, accounts for about 90% of esophageal cancer cases globally, while esophageal adenocarcinoma is more prevalent in Western countries (4–6).

The therapies for ESCC include surgical resection, chemotherapy, radiotherapy, targeted therapy, and immunotherapy. For the early stage of ESCC, surgical resection is a definitive treatment. However, most patients have a poor prognosis because of the late onset of symptoms and diagnoses (7–10). Recently, we achieved a better therapeutic efficacy through targeted arterial infusion of verapamil combined with chemotherapy (TVCC) in a cohort of 46 patients with advanced ESCC (stage III and IV). TVCC reduced tumor stage significantly, improved the survival time, and achieved an 89.13% overall effective rate (complete and partial response; ref. 11).

Verapamil is an L-type voltage-gated calcium channel blocker binding to the subunit receptor in the plasma membrane, and it is a well-established drug strategy for heart arrhythmia, angina, and hypertension through antagonism of calcium influx (12–14). P-glycoprotein mediates the efflux of chemotherapeutic drugs from cells (15, 16). Verapamil, as a P-glycoprotein inhibitor, can obstruct the efflux of anticancer agents, thereby raising the intracellular concentration of chemotherapeutic agents in cancer cells (17, 18). TVCC has been widely used in the clinical treatment of diverse cancers, such as metastatic colorectal cancer (19), lung cancer (20), liver cancer (21, 22), and gastric cancer (23). However, the major challenge to cancer treatment is the development of multidrug resistance (MDR), which can cause relapse or poor prognosis (24–26).

Although the genetic and epigenetic landscapes of ESCC have been comprehensively elucidated recently and provide deep insights into the initiation and progression of ESCC (27–33), including cell cycle regulation (*TP53*, *RB1*, *CDKN2A*, *FBXW7*, *NFE2L2*, *ADAM29*, *FAM135B*, *CCND1*), the Hippo and Notch pathways (*YAP1*, *AJUBA*, *NOTCH1*, *NOTCH2*, *NOTCH3*), RTK/MAPK/PI3K signaling (*PIK3CA*, *FGFR1*, *ERBB2*), microRNAs (miR-548k, miR-21, miR-34b, miR-92a), and epigenetic modification (*MLL2*, *ASH1L*, *MLL3*, *SETD1B*, *CREBBP*, *EP300*, *KDM6A*; refs. 27, 34–43), the underlying molecular mechanisms of MDR in clinical therapies are yet to be fully elucidated.

Genetic heterogeneity is a fundamental property of cancers and can contribute to the development of drug resistance or diverse clinical outcomes due to genetic instability or alterations (44–47). Beneficial new mutations under selective pressure can drive subclonal expansion, representing various tumor evolution models, such as punctuated evolution, branching evolution, linear evolution, and neutral evolution, leading to acquired resistance and relapse of cancer during therapeutic interventions (46, 48–50). In addition, epigenetic heterogeneity also plays an important role in the emergence of MDR (51, 52). Epigenetic patterns have great plasticity and vary dramatically as therapies proceed and the tumor develops.

Despite the advances of molecular etiology and target therapies, there is a paucity of evidence about the molecular mechanisms, genetic and epigenetic heterogeneity, and clonal evolutionary trajectories of MDR in TVCC treatments. Here, we address these critical issues through comparative and integrative investigation of genomic and epigenomic characteristics on serial specimens obtained from 7 patients with ESCC with TVCC treatment. We enrolled a cohort of 7 patients with ESCC from 3 groups: complete response (CR), partial response (PR), and progressive disease (PD). Serial specimens were obtained at every cycle of therapeutic intervention and conducted with whole-exome sequencing (WES) with matched tissues. In a subset of this cohort, 1 case from each group was subjected to whole-genome bisulfite sequencing (WGBS). We revealed the dynamics of temporal genetic and epigenetic heterogeneity and clonal evolutionary processes during combined therapies. Notably, we observed a predominant subclonal population, implicating acquired resistance, and we identified potentially novel MDR genes, which may facilitate personalized treatment and the development of new therapeutic targets to reverse MDR in ESCC.

## Results

### Treatment-specific alterations of molecular landscape.

**Figure 1:**
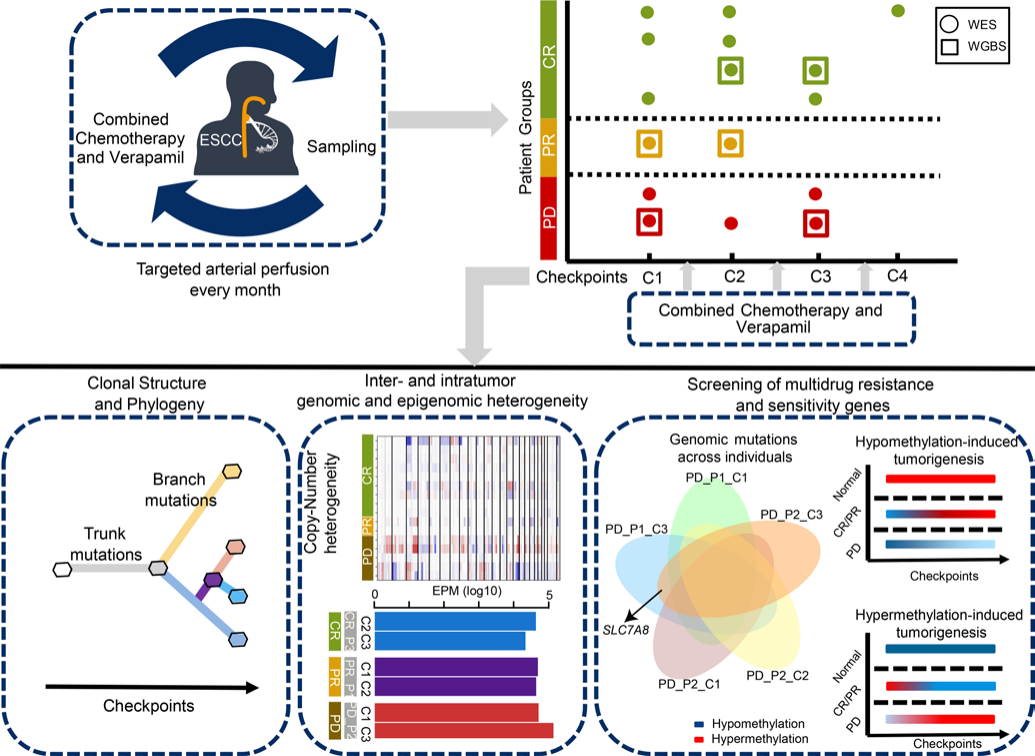
Schematic representation of patient sampling and analytic strategy during therapies. Seven patients with ESCC (*n* = 7) were treated with TVCC every month. Patients were divided into 3 groups: complete response (CR), partial response (PR), and progressive disease (PD). We collected 16 samples at every checkpoint of therapeutic intervention and performed whole-exome sequencing (circle) with matched tissues. Whole-genome bisulfite sequencing (square) was conducted for 3 patients from each group (CR, PR, and PD). The bottom panel shows comparative and integrative investigation of genomic and epigenomic characteristics.

**Figure 2:**
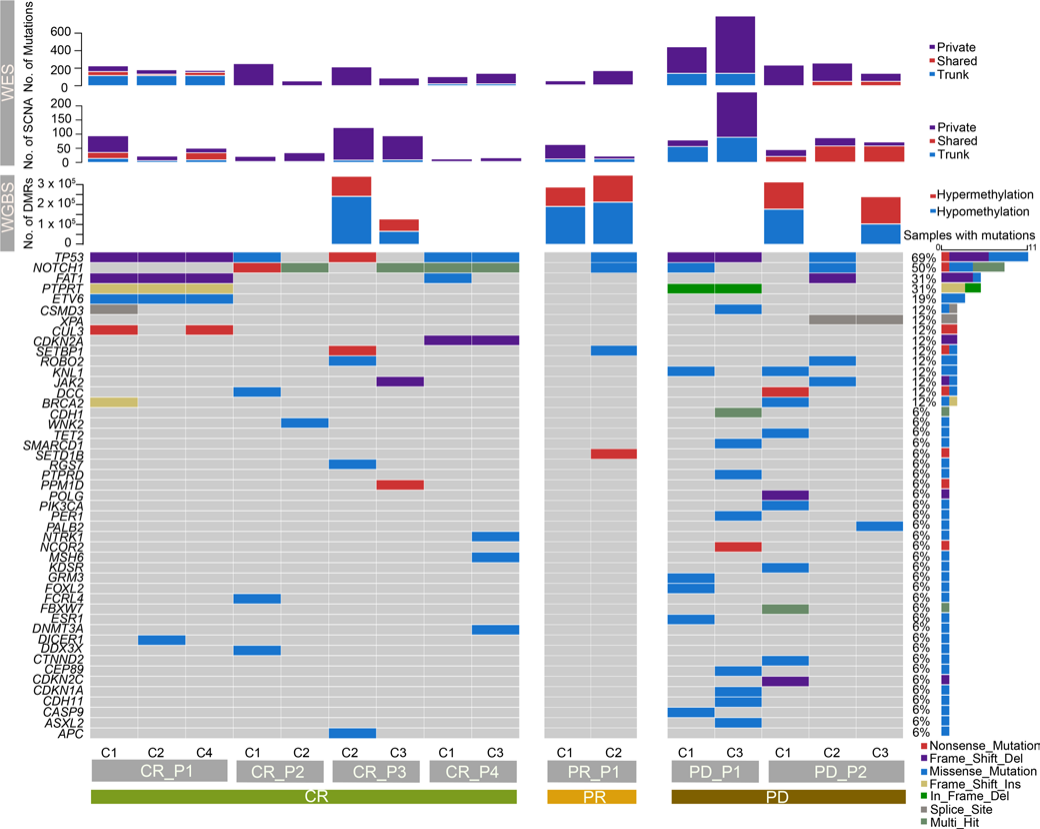
Profiles of molecular alternations due to treatments. Each column represents a sample obtained from serial checkpoints under combined therapies. The top 3 panels depict a specific molecular landscape, including the number of somatic mutations, somatic copy number aberrations (SCNAs), and differentially methylated regions (DMRs). The bottom panel shows somatic driver mutations in different groups. Mutation frequencies are displayed on the right.

TVCC was performed once a month in 7 patients with ESCC, 1 to 3 times per patient, and specimens were obtained at every checkpoint (Figure 1). In this cohort, 4 patients achieved CR, 1 patient showed PR, and 2 patients had PD (see Supplemental Table 1; supplemental material available online with this article; https://doi.org/10.1172/jci.insight.150203DS1). We performed WES on 16 serial specimens from 7 patients with ESCC, with a median sequencing depth of 173× for tumors and 88× for normal samples (Supplemental Figure 1A and Supplemental Table 2). Meanwhile, 6 serial specimens from 3 cases were subjected to WGBS, 1 case from each group, with a median sequencing depth of 35× for tumors and normal samples (Supplemental Figure 1B and Supplemental Table 3). In total, we identified 2053 nonsilent mutations and 661 silent mutations (Supplemental Table 4 and Supplemental Table 5). The PD group showed the most abundant somatic mutations and somatic copy number aberrations (SCNAs; Figure 2 and Supplemental Figure 2, Kruskal-Wallis rank-sum test, *P* = 0.041). Considerable variations were observed, as expected for different clinical outcomes. The number of somatic mutations and SCNAs decreased in the CR group during the serial therapies of TVCC, whereas the PD group exhibited a trend toward increased mutations. Remarkably, the PR patient demonstrated a substantial increment of somatic mutations and an opposite change in SCNAs. In methylation profiles, we also found a substantial reduction of the number of differentially methylated regions (DMRs) in the CR group and a trend toward rising DMRs in a PR patient during therapeutic interventions; the PD patient demonstrated a lower number of DMRs at checkpoint 3 but an elevated number of SCNAs, indicating the important role of SCNAs in the progression of the tumor.

We next investigated the dynamic patterns of somatic mutations in ESCC-related genes during combined therapies and identified 75 driver mutations based on previously reported criteria (Supplemental Table 6 and refs. 53, 54), including several well-known ESCC-implicated genes, such as *TP53*, *NOTCH1*, *FAT1*, *CDKN2A*, and *PIK3CA* (Figure 2). Evolutionary characteristics of driver events occurred differently in the CR, PR, and PD groups. *TP53* alterations were subjected to early events in patients CR_P2 and CR_P3 and disappeared during tumor therapies. *CSMD3* and *BRCA2* occurred early in patient CR_P1. On the contrary, patient PR_P1 revealed late alterations in *TP53* and *NOTCH1*. Similarly, patient PD_P1 developed more late mutations during the treatments, in accordance with a previous study (55). This finding uncovers the impact of genetic heterogeneity on clinical consequences and suggests the evolution of driver mutations under therapeutic selection pressure.

### Dynamics of temporal intratumoral heterogeneity during treatments.

Intratumoral heterogeneity (ITH) has a close relationship with clinical outcomes and tumor evolution (53, 56). To assess the dynamics of temporal ITH during TVCC, we analyzed how the dynamics of mutational processes, burden of mutations, and SCNA profiles contributed to ITH during treatments.

**Figure 3:**
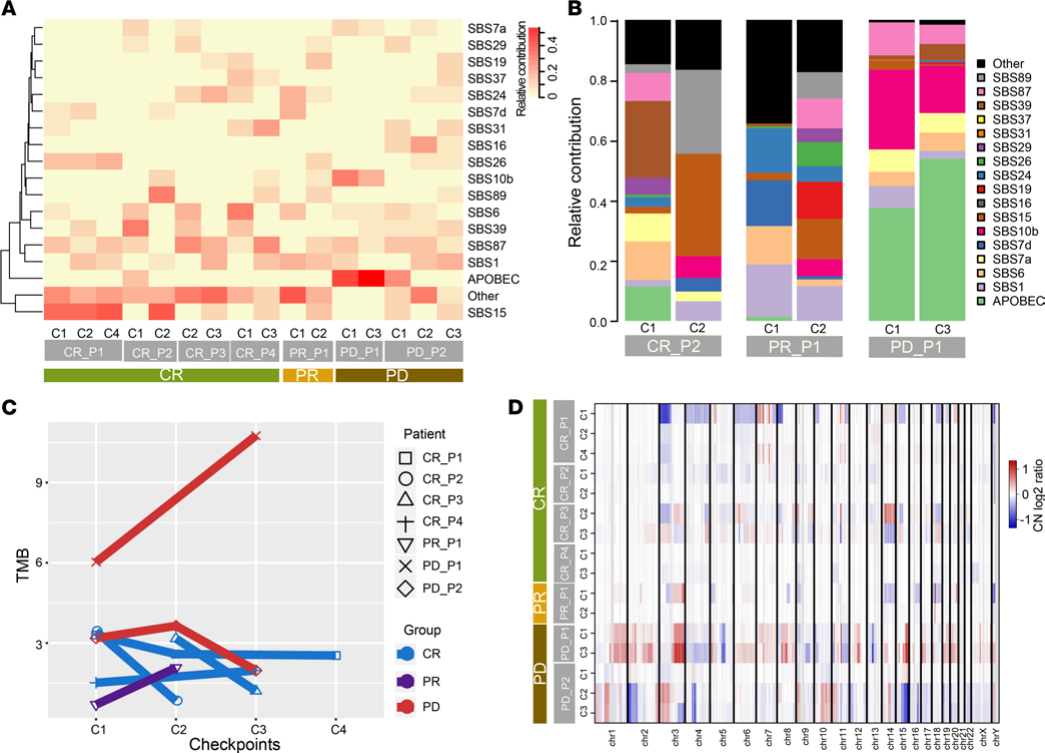
Characteristics of temporal genomic heterogeneity. (**A**) Mutation signatures detected in each ESCC sample. (**B**) Changes in relative mutation signature contribution between pretreatment and treatment stages in CR_P2, PR_P1, and PD_P1. (**C**) Changes in mutation burden in 3 groups during treatments. (**D**) SCNA profiles of amplifications (red) and deletions (blue) based on log_2_ ratio of copy number. Tumor samples are arranged in rows and columns represent genomic positions.

APOBEC signature (cytidine deamination) was the predominant mutational process in ESCC, as reported in previous studies (54, 57). We observed a significant accumulation of the APOBEC signature in patient PD_P1 but a reduction in CR_P2 (Figure 3, A and B). Mutational signature 39 (of unknown etiology) demonstrated a decreased contribution in the CR group as opposed to the PD group, which was elevated. Moreover, the change in defective DNA mismatch repair contribution, including signatures 6, 15, and 26, varied among the CR patients. Signatures 15 and 26 were increased in patients CR_P1 and CR_P2 (Figure 3A), but signature 6 was reduced in patients CR_P3 and CR_P4 (Supplemental Figure 3A). Combined therapies led to decreased abundance of mutational signatures in patient CR_P2 and markedly improved mutational signatures in patients PR_P1 and PD_P2 (Figure 3B and Supplemental Figure 3A). A higher mutational burden was observed in the PD group than the CR group (5.11 vs. 2.28), which had a lower mutational burden, and the PR patient showed a trend toward increased mutations during therapies (Figure 3C and Supplemental Figure 3B, Kruskal-Wallis rank-sum test, *P* = 0.031). This finding potentially reflects enormous differences in the genetic heterogeneity of patients with ESCC and illustrates the dynamics of inter- and intratumoral heterogeneity during clinical treatments.

The PD patients represented the most SCNAs, and amplifications accounted for the major portion. The proportion of amplifications of PD_P1 and PD_P2 was 85.14% and 62.38%, respectively (Figure 3D and Supplemental Table 7). However, substantial variation in evolution lineages occurred in these 2 patients. Trunk SCNAs in PD_P1 were predominantly in the early stage (checkpoint 1), and more private SCNAs emerged in the late stage because of therapeutic pressure. In contrast to PD_P1, patient PD_P2 had fewer SCNAs and had more shared SCNAs in late checkpoints (Figure 2 and Figure 3D). Only 8 common SCNAs were identified in PD patients (Supplemental Table 8), including a well-known oncogene *NRG1* in non–small cell lung cancer, pancreatic cancer, and invasive mucinous adenocarcinoma (58, 59). These results suggest strong intertumoral heterogeneity and ongoing SCNA evolution during treatments.

### Clonal evolutions under combined therapies.

**Figure 4:**
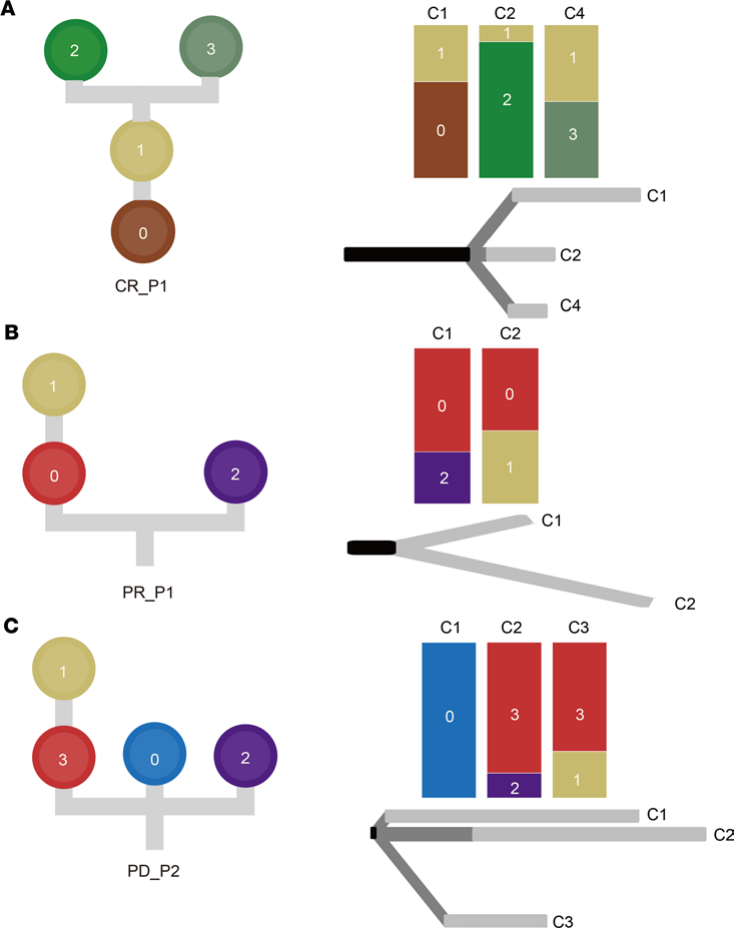
Heterogeneity of clonal evolutions under combined therapies. Phylogenetic trees show the evolution of clone or subclone at the left side of each panel. The top right of each panel demonstrates the dynamics of clonal composition at each checkpoint across every sample under selective pressures. A discrete-characters parsimony method was used to generate phylogenetic relations based on somatic mutations and copy number aberrations and is shown at the bottom right of each panel. Black: trunk, dark gray: shared branches, gray: private branches. Diverse evolutionary paths were inferred across patients: (**A**) linear evolution (CR_P1), (**B**) unrooted branching evolution (PR_P1), and (**C**) divergent evolution (PD_P2).

To decipher clonal evolution and ongoing selective pressure toward therapeutic intervention, we inferred clonal populations and reconstructed phylogenetic trees based on somatic mutations and SCNAs. We observed various models of evolutionary dynamics, including linear, divergent, and branching evolution (Figure 4). In the CR group, patient CR_P1 exhibited a linear evolution, where clone 0 evolved as a dominant clone and subclone 1 derived different descendent subclones under selective pressures. Furthermore, checkpoint 4 had fewer private mutations (Figure 4A). Patients CR_P2 and CR_P3 showed a divergent evolution (Supplemental Figure 4A and Supplemental Figure 4B). Different branches existed in continuous checkpoints during therapies and displayed a similar trend toward the change in the number of private mutations. Patient CR_P4 contained 2 independent clones at pretreatment checkpoint 1, and clone 0 disappeared at checkpoint 3. Meanwhile, clone 2 evolved subclone 1 during TVCC exposure (Supplemental Figure 4C). Similarly, patient PR_P1 demonstrated the same pattern of unrooted branching evolution (Figure 4B) but obtained a resistant subclone 1 in late stage, which led to a different clinical outcome compared with CR_P4. Patient PD_P1 showed a rooted branching evolution. A potential resistant clone 2 was already present in the early stage (checkpoint 1) and was retained with its descendent subclone 0 after the therapies (Supplemental Figure 4D). In contrast, patient PD_P2 exhibited a divergent evolution and evolved a dominated and resistant clone 3, then a descendent subclone 1 during the treatments (Figure 4C). Taken together, this result unveiled diverse evolutionary trajectories in patients with various clinical outcomes and may explain different evolutionary paths of drug resistance across patients.

### Acquired resistance gene SLC7A8.

**Figure 5:**
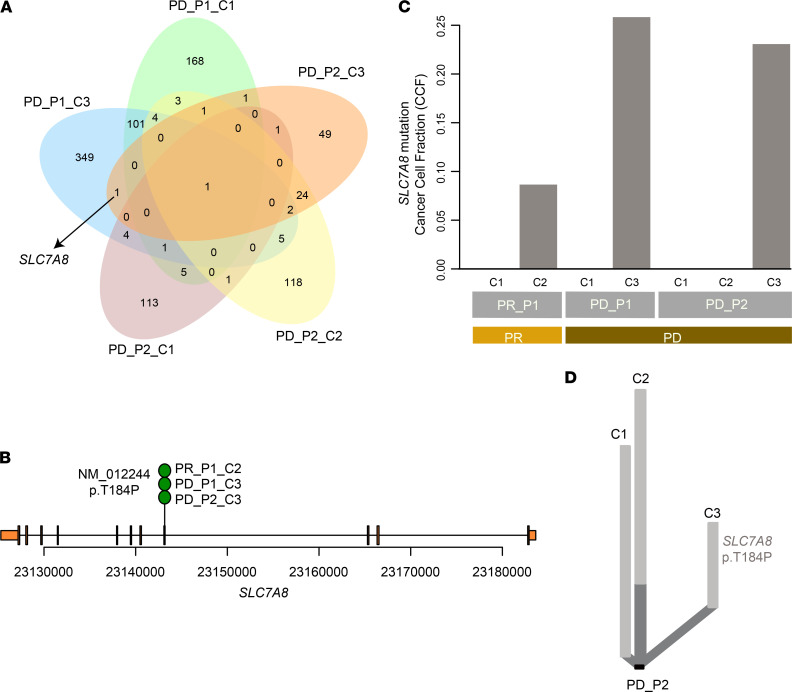
Acquired resistance mutation of *SLC7A8*. (**A**) Venn diagram of acquired mutation in PR and PD patients. (**B**) Schematic of *SLC7A8* somatic mutation in this ESCC cohort. Only 1 mutation was identified from treatment-specific samples. (**C**) Cancer cell fraction (CCF) of *SLC7A8* p.T184P across PR and PD patients. (**D**) Private branch contained *SLC7A8* p.T184P and is illustrated in the phylogenetic tree of patient PD_P2.

To gain insight into the mechanisms of therapy resistance, we investigated the common mutated gene after therapies in PD patients (Figure 5A). A missense mutation, *SLC7A8* p.T184P, was not detected in pretreatment samples, which occurred concurrently at treatment-specific checkpoints of patients PD_P1 and PD_P2. Furthermore, it was also detected at the late stage (checkpoint 2) of patient PR_P1 (Supplemental Figure 5 and Figure 5B). We estimated the cancer cell fraction of *SLC7A8* p.T184P for each patient during the treatments, which was higher in PD patients (PD_P1_C3: 0.26, PD_P2_C3: 0.23) than in the PR patient (PR_P1_C2: 0.09; Figure 5C). Additionally, it was a private mutation in all PR and PD groups (Supplemental Table 5 and Figure 5D) and evolved as a subclone (PR_P1, subclone 1; PD_P1, subclone 0; PD_P2, subclone 1; Figure 4, B and C and Supplemental Figure 4D) upon verapamil treatment, indicating that the hotspot mutation in *SLC7A8* might play an important role in resistance to TVCC.

**Figure 6:**
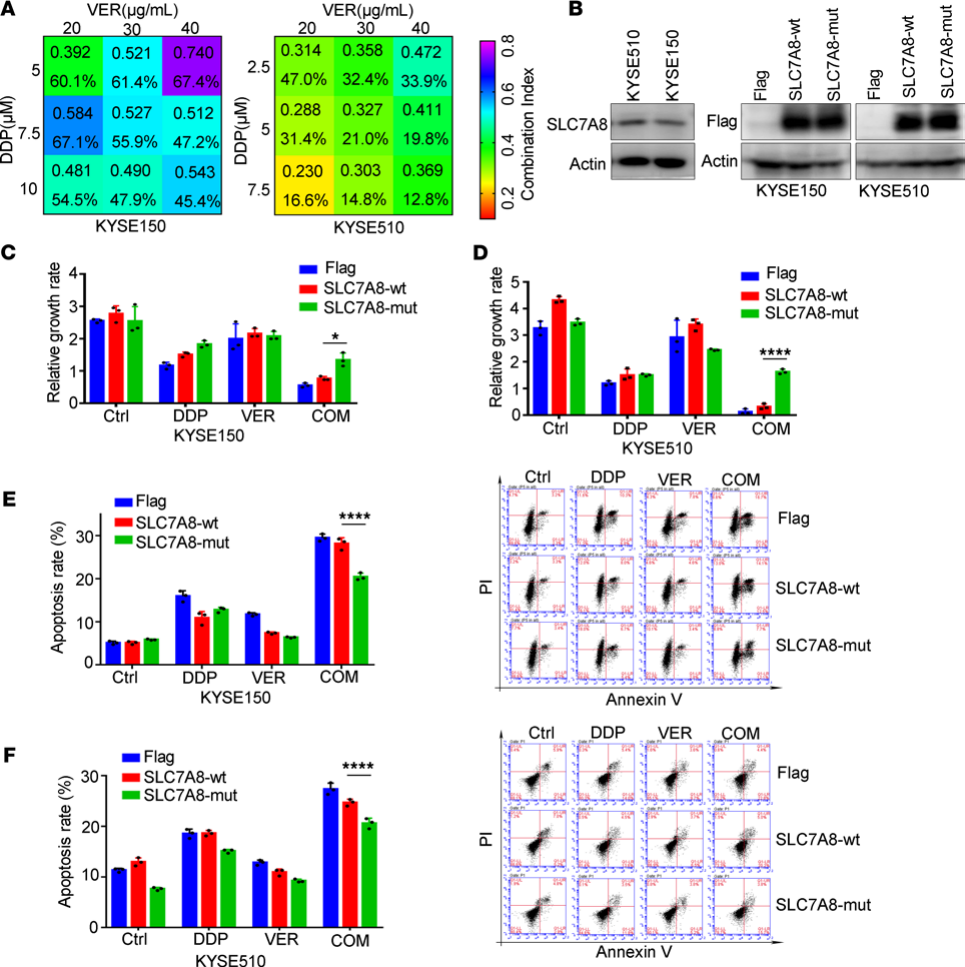
Functional validation of *SLC7A8* mutation in multidrug resistance. (**A**) Combination index and cell survival rate (%) of cisplatin and verapamil. (**B**) Immunoblotting analysis of endogenous expression of SLC7A8 (left panel) and the forced overexpression of *SLC7A8*-WT versus mutant *SLC7A8* (*SLC7A8*-mut) in KYSE150 and KYSE510 cell lines (right panel). Cell viability measurement revealed that *SLC7A8*-mut cells represented higher cell growth rate than *SLC7A8*-WT cells in KYSE150 (**C**) and KYSE510 (**D**) cell lines. PI staining flow cytometry showed the apoptotic effect of *SLC7A8*-WT and *SLC7A8*-mut in KYSE150 (**E**) and KYSE510 (**F**) cell lines. KYSE150 cell was treated with 10 μM cisplatin and 20 μg/mL verapamil. KYSE510 cell was treated with 2.5 μM cisplatin and 20 μg/mL verapamil. Three independent experiments were performed. *P < 0.05, ****P < 0.0001. Data represent mean ± SD. Two-way ANOVA followed by Tukey’s multiple-comparison test was used. VER, verapamil; DPP, cisplatin; Ctrl, control; mut, mutation; COM, combination; PI, propidium iodide.

To discover the biological function of mutant *SLC7A8* (*SLC7A8*-mut), we used the KYSE150 and KYSE510 cell lines, which have been proven to be the least sensitive to cisplatin (60), for further research. The acquired resistance mutation *SLC7A8* p.T184P during TVCC was not observed in KYSE150 and KYSE510 cell lines based on whole-genome sequencing profiling of these 2 cell lines (Supplemental Tables 9 and 10). We first assessed the IC_50_ of verapamil in KYSE150 and KYSE510 by MTS assay after 72 hours of treatment (KYSE150, 77.45 μg/mL, 95% CI: 74.33–80.7 μg/mL; KYSE510, 59.22 μg/mL, 95% CI: 55.37–62.88 μg/mL). A proper concentration of cisplatin (from 2.5 μM to 10 μM) and verapamil (from 20 μg/mL to 40 μg/mL) was selected to treat the cells, and the combination index was determined by the Chou-Talalay method. A combination index value less than 1 was observed in KYSE150 and KYSE510 with combination treatment of cisplatin and verapamil, indicating a remarkable synergistic effect (Figure 6A). Notably, low-dose cisplatin and verapamil displayed strong synergy with a combination index value less than 0.5, which suggested that verapamil efficiently enhanced the cytotoxic effect of cisplatin. To explore the biological function of mutant *SLC7A8* in chemotherapy, we detected the endogenous expression of SLC7A8 and constructed stably expressing *SLC7A8*-WT and mutant *SLC7A8* KYSE150 and KYSE510 cell lines (Figure 6B). We further determined how cell viability was altered by cisplatin and verapamil treatment. We treated cells with cisplatin and verapamil and measured the cell growth rate at 48 hours compared with 0 hours. The cell viability of empty vector, *SLC7A8*-WT, and *SLC7A8*-mut cells significantly decreased in the cotreatment group (Figure 6, C and D). However, the *SLC7A8*-mut cells had a higher cell growth rate than *SLC7A8*-WT cells in the cotreatment group, which demonstrated that *SLC7A8*-mut might reduce the cytotoxicity of combination therapy. Next, we performed propidium iodide (PI) staining flow cytometry to detect the apoptotic effect in the *SLC7A8*-WT and *SLC7A8*-mut group after 48 hours of cotreatment. As shown in Figure 6, E and F, the cell apoptosis induced by cisplatin and verapamil cotreatment was more significant than that induced by single drug treatment. Nevertheless, compared with *SLC7A8*-WT, the *SLC7A8*-mut group had a decreased apoptotic rate. These results implied that *SLC7A8*-mut blocked the therapeutic effect of the cisplatin and verapamil combination. In summary, cisplatin and verapamil exerted synergistic inhibitory effects on ESCC cells, and *SLC7A8*-mut endowed cells with resistance to the cisplatin and verapamil combination treatment. Taken together, this finding indicates the important role of acquired *SLC7A8* mutation in resistance to TVCC treatment.

### Patterns of epigenetic dynamics.

**Figure 7:**
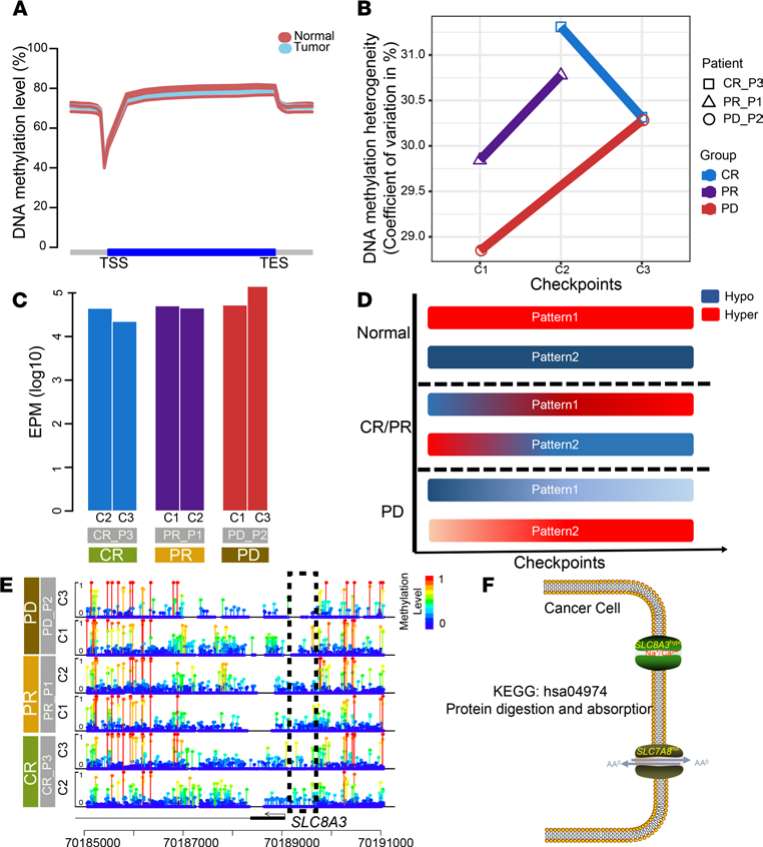
Epigenetic heterogeneity and dynamics. (**A**) Distribution of global CpG methylation levels across gene bodies in tumor and normal samples. TSS, transcription start site; TES, transcription end site. (**B**) The coefficient of variation was calculated to assess intertumor DNA methylation heterogeneity per patient. (**C**) Epiallele shifts per million loci (EPM) in each sample. (**D**) Two patterns depict the dynamics of methylation level under treatments. Pattern 1 assumed that hypomethylation level of promoter regions facilitated MDR and tumorigenesis, and pattern 2 assumed that hypermethylation promoted the evolutionary processes of MDR. (**E**) Lollipop plot shows the CpG methylation level of *SLC7A8* promoter region for each patient. (**F**) Altered pathway (protein digestion and absorption) composed of mutant *SLC7A8* and promoter hypomethylation of *SLC8A3*. KEGG, Kyoto Encyclopedia of Genes and Genomes.

Epigenetic alterations have been reported to be associated with tumorigenesis and drug resistance in previous tumor studies (32, 61, 62). To decipher epigenetic ITH in ESCC and the potential relationship between epigenetic dynamics and MDR, we performed WGBS for 6 samples that were obtained from 3 patients: a CR, PR, and PD patient. The global methylation levels were lower in tumor tissues compared with normal tissues (Figure 7A), as described previously (61). The coefficient of variation was calculated to assess intertumoral heterogeneity of DNA methylation levels across patients (63). The CR group displayed a trend toward declining DNA methylation levels in contrast to the PR and PD groups (Figure 7B), suggesting substantial variations of clinical outcomes on the basis of epigenetic heterogeneity among patients with ESCC. Moreover, we used EPM (epiallele shifts per million loci) to evaluate the epiallele shift of epigenetic landscape between different stages of therapies. The degree of EPM was highest in patient PD_P2 and demonstrated an upward trend at checkpoint 3 (Figure 7C and Supplemental Table 11).

To elucidate the epigenetic dynamics during therapies, we raised 2 hypotheses to depict the changes in methylation level during treatment. The first hypothesis (pattern 1) assumed that the hypomethylation level of promoter regions facilitated MDR and tumorigenesis. In contrast, the second hypothesis (pattern 2) assumed that hypermethylation promoted the evolutionary processes of MDR (Figure 7D). We observed 11 genes in concordance with pattern 1, including 8 protein-coding genes, such as *PZP*, *FKBPL*, and *SLC8A3* (Figure 7E). Only 2 long noncoding RNAs (*AL391415.1* and *LINC01509*) were detected according to pattern 2 (Supplemental Table 12). Interestingly, *SLC7A8* and *SLC8A3* were enriched in the common pathway of protein digestion and absorption (Figure 7F), suggesting a potential relationship between alteration of amino acid metabolism and MDR.

## Discussion

Cisplatin-based chemotherapy is recommended as the first-line treatment for advanced ESCC, but only a small proportion of patients have complete or partial response to chemotherapy. Substantial clinical efficacy of TVCC has been reported by us in several clinical trials, including colorectal cancer, gastric cancer, lung cancer, and liver cancer trials. In this study, we adopted this clinical approach in ESCC and achieved good clinical results. Nevertheless, the MDR phenomenon was observed in some patients. Therefore, it is important to elucidate intrinsic and acquired resistance mechanisms that arise during combined therapies.

To gain insight into the temporal dynamics of genomic alterations and clonal evolution toward therapy resistance, we performed a comprehensive genomic and epigenomic profiling of ESCC specimens from 7 patients, including pretreatment- and treatment-specific samples, to reveal the underlying mechanisms of MDR. We observed the accumulation of molecular alterations in PD patients and a trend toward declining molecular alterations in CR patients. In addition, diverse temporal inter- and intratumoral heterogeneity were also found in patients with different clinical outcomes. Various mutational processes were observed in patients with different clinical outcomes. The cytidine deamination process, APOBEC signature, attributed to the activation of AID/APOBEC cytidine deaminases, is the predominant mutational signature in ESCC, as reported in our previous studies (57, 64). We revealed a substantial accumulation of the APOBEC signature in PD_P1 but a trend toward diminishing APOBEC signature in CR_P2. Intertumoral heterogeneity might result in diverse clinical responses (65), and adaptive mutagenesis (increased mutation generation by therapy induction) can accelerate the emergence of resistant cancer cells. Besides APOBEC signature, defective DNA mismatch repair signatures, including SBS 6, 15, and 26, presented opposite patterns in the CR group. DNA mismatch repair mediates cell cycle arrest and apoptosis and participates in DNA metabolic pathways (66). The alteration of signature abundance during therapies can lead to different clinical responses and fuel a shift in treatment paradigm, thus improving clinical outcomes. High genetic heterogeneity driven by accumulating genetic instability and alterations might lead to acquired resistant clones under selective pressure imposed by combined therapies, and the outgrowth of resistant clones will expand during therapies. Furthermore, we detected various clonal patterns, including linear, divergent, and branching evolution, during therapies. Intriguingly, we detected an acquired resistance mutation, *SLC7A8* p.T184P, as a hotspot mutation, in PR and PD patients. Meanwhile, the evolutionary dynamics of subclones carrying the *SLC7A8* mutation implied the emergence of acquired resistance. Further functional assays showed that the mutant *SLC7A8* reduced the cytotoxicity of combination therapy, as demonstrated by a higher cell growth rate and a decreased apoptotic rate.

Besides the genetic heterogeneity contributing to phenotypic divergences in tumors, epigenetic mechanisms can also result in various characteristics in cancer cells. It has become evident that inter- and intratumoral epigenetic heterogeneity can predispose patients to various clinical outcomes and foster resistance to treatments (67, 68). We profiled epigenomic alterations from global methylation analysis and found a higher level of epigenetic heterogeneity in PD patients. Notably, we demonstrated hypomethylation status in promoter regions of 8 protein-coding genes in PD patients compared with CR patients on the basis of epigenetic dynamic patterns during therapies. An enriched pathway, protein digestion and absorption, including *SLC7A8* and *SLC8A3*, highlighted the potential role of amino acid metabolism in MDR. Yasuhiro et al. reported that the overexpressed *LLGL2* in estrogen receptor–positive breast cancer promoted leucine uptake by upregulated *SLC7A5* to induce tamoxifen resistance (69). However, our conclusions are limited by the small sample size; further studies are needed to better understand clonal evolution involved in therapeutic resistance and reveal the potential mechanisms of amino acid metabolism in MDR of TVCC in ESCC.

In summary, integrative analysis of the genomic and epigenomic landscape may enhance the understanding of evolutionary processes of acquired resistance and unveil novel therapeutic targets to reverse MDR in clinical treatments.

## Methods

### Sample collection.

Patients who had no contraindication for verapamil, cisplatin, lobaplatin, or 5-ﬂuorouracil underwent esophageal endoscopy, computed tomography, and pathological examination to confirm the histologic grade of ESCC and lymph node metastasis. Seven patients in 3 groups, CR, PR, and PD (19, 20, 23), were recruited, and they provided documented informed consent (Supplemental Table 1). Response evaluation criteria were defined as follows: a) CR — complete disappearance of target lesions in the esophageal endoscopic examination and barium x-ray, disappearance of lymphatic and distant metastases disappeared; the effect lasted for at least 1 month; b) PR — 30% or more reduction of the maximum diameter of target lesions in the barium x-ray and esophageal endoscopic examination; lymphatic and distant metastases declined by more than 30% for more than 1 month since the treatment started; c) PD — at least a 20% increase in bidimensional diameter or new lesions appeared. The Seldinger technique was used to conduct TVCC for each patient monthly. Tumors and paired normal specimens were obtained from each patient at every checkpoint of therapeutic intervention.

### WES and WGBS.

DNA was extracted using QIAamp DNA Micro kit (QIAGEN) according to the manufacturer’s instructions. The qualified genomic DNA was sheared into fragments of 200–300 bp by a Covaris focused ultrasonicator. Then, the fragments were end-repaired, A-tailed, and ligated with adapters. For WGBS library generation, DNA fragments were ligated with cytosine methylated barcodes. Bisulfite conversion was conducted using EZ DNA Methylation-Gold Kit (Zymo Research). Selective DNA fragments were amplified by PCR. SureSelect Human All Exon V6 kit (Agilent Technologies) was used to capture the human exonic regions. High-throughput sequencing was performed on a Hiseq X Ten platform to generate 150 bp paired-end reads. The raw sequencing data have been deposited in the Genome Sequence Archive (70) in National Genomics Data Center (71), China National Center for Bioinformation/Beijing Institute of Genomics, Chinese Academy of Sciences, under accession number HRA000936 (publicly accessible at https://ngdc.cncb.ac.cn/gsa).

### Somatic mutations and copy number aberration detection.

Quality control was performed by Cutadapt (v1.19; ref. 72) and TrimGalore (v0.5.0) (http://www.bioinformatics.babraham.ac.uk/projects/trim_galore/) to remove adapters, low-quality segments, and discarded reads shorter than 50 bp. High-quality reads were aligned to human reference genome (GRCh38.p12) using BWA (v0.7.17; ref. 73) to generate a binary alignment map (BAM) format file. Duplicated reads were marked and removed by Picard (v1.112) (https://broadinstitute.github.io/picard/). Somatic single-nucleotide variants/InDels were detected by GATK (v4.1.0.0, Mutect2; ref. 74) and followed by ANNOVAR (Version 2018Apr16) (75) to functionally annotate somatic variants. We applied the R package maftools (v2.0.10; ref. 76) to classify and visualize variants and rank mutated genes. We set the following criteria to filter low-quality or possible false somatic variants: a) coverage of mutated sites should be more than 10 reads and at least 4 reads harboring mutations in the tumor sample, with at most 2 reads with mutations in the corresponding normal samples; b) filter common SNPs in dbSNP150, Eastern Asia of 1000 Genomes, ExAC, gnomADe, and ESP6500. SCNAs were assessed by Control-FREEC (v11.5; ref. 77). The trunk was defined as the ubiquitous mutations that occurred in all tumor samples, shared branches representing mutations that existed in partial samples, and private branches representing mutations merely present in 1 sample (54).

### Identification of driver mutations.

Putative cancer driver genes were curated from previously reported ESCC-implicated genes (27, 34–36, 54, 57, 64, 78–81), pan-cancer analysis of 21 tumor types (82), and COSMIC cancer gene census (v92; ref. 83). Nonsilent mutations located within these genes were selected based on the following criteria: a) recessive genes annotated by COSMIC, and the mutation was deemed as deleterious; for example, stop-gain, splicing mutations, frameshift insertions and deletions, and nonsynonymous variants predicted deleterious by 2 of 3 approaches, including SIFT (84), Polyphen2 (85), and MutationTaster (86); b) an exact match or at least 3 COSMIC variants located within 15 bp flanking regions of the mutation (53, 54).

### Mutational signature analysis.

To characterize the underlying mutational processes during combined therapies, we used SignatureAnalyzer (87, 88), a BayesNMF algorithm, and MutationalPatterns (89), a non-negative constrained least-squares algorithm, to estimate the relative contribution of COSMIC (v3.1; ref. 90) signatures.

### Clonality inference and phylogenetic tree construction.

We estimated tumor clonality and cellular prevalence by incorporation of variant allele frequency and allele-specific copy number of the genomic region at each mutation using PyClone (v0.13.0) (91) based on a Bayesian clustering method. The construction of clonal evolution was inferred by CITUP (92) in the QIP version. Mutations should satisfy the infinite-sites model, which means that a mutation occurs only once during the evolutionary process. To infer clonal evolution, 4 evolutionary constraints are considered: a) sum rule, which states that the sum of cellular prevalence over all descendant subclones should be equal or less than the ancestor clone in each sample; b) crossing rule, in which the cellular prevalence of a descendant subclone must be smaller than the ancestor clone; c) connectivity rule, which states that a subclone is connected to only 1 clone; d) pigeonhole principle, indicating that if the sum of cellular prevalence of 2 clusters exceeds 100% in a single sample, these 2 clusters will be considered dependent and have an evolutionary relationship (53, 55). In addition, we also constructed phylogenetic trees using discrete-characters parsimony method implemented in PHYLIP (v3.697; ref. 93) based on a binary matrix generated by somatic mutations and copy number aberrations.

### Identification of DMRs and annotation.

We aligned the WGBS reads to human reference genome (GRCh38.p12) using BS-Seek2 (v2.1.8; ref. 94) and Bismark (v0.22.1) (95) after removed adapters and low-quality reads with default parameters. DMRs were detected by CGmapTools (v0.1.2) (96) and fulfilled the following criteria: a) *P* value less than 0.05 and b) the difference in methylation level between 2 samples was greater than 0.15. The comprehensive gene annotation Release 30 in GTF was downloaded from GENCODE (https://www.gencodegenes.org/human/) on the basis of the GRCh38.p12 reference genome. CpG islands were downloaded from the University of California Santa Cruz (http://genome.ucsc.edu/). The 2000 bp flanking regions of CpG islands were defined as CpG shores and the same as CpG shelves (2000 bp flanking regions of CpG shores). The 15-state ChromHMM annotation of esophagus tissue E079 was downloaded from Roadmap Epigenomics (97) to mark regulatory elements.

### Epigenetic dynamics during therapeutic interventions.

We detected epiallele shifts in each eloci between tumors and paired normal samples at 4 adjacent CpGs in 16 patterns by methclone (v0.1; ref. 98), where “0” represented an unmethylated CpG and “1” stood for a methylated CpG. It was defined as a significant change between foreground and background if the combinatorial entropy difference was ΔS less than –70.

Epigenetic dynamics in promoter regions were compared simultaneously between tumors and paired normal samples, 2 serial tumor samples at adjacent checkpoints, and different clinical groups by in-house scripts. Based on the changes in methylation level, we raised 2 hypotheses to depict the epigenetic dynamics during treatment. First, the hypomethylation level of promoter regions facilitated MDR and tumorigenesis (pattern 1). Second, hypermethylation promoted the evolutionary processes of MDR (pattern 2). According to these 2 hypotheses, candidate genes with epigenetic alterations in promoter regions were identified.

### Cell culture.

ESCC cell lines KYSE150 and KYSE510 were gifts from Y. Shimada of Kyoto University, Kyoto, Japan. They were authenticated using short tandem repeat profiling. Cells were cultured in RPMI 1640 (Gibco) supplemented with 10% FBS (Gibco) in a 5% CO_2_ humidified incubator at 37°C.

### Cell viability assay.

Cells were seeded in 96-well plate at the density of 3 × 10^3^/well for 24 hours. Old culture medium was removed and 100 μL 10% MTS was added for 1 hour, and the 490 nm absorbance was measured with Infinite M200 PRO (Tecan). The drug combination was determined by the combination index method (CalcuSyn software, Biosoft) according to the median-effect analysis of Chou and Talalay (99). A combination index less than 1 indicates synergy, greater than 1 indicates antagonism, and equal to 1 indicates an additive effect.

### Flow cytometry analysis.

Cells were seeded in 6-well plate and treated with cisplatin or verapamil or combined cisplatin and verapamil. The annexin V-FITC apoptosis detection kit (Beyotime) was used to detect the apoptotic cells according to the manufacturer’s instructions. The samples were analyzed by Accuri C6 (BD Biosciences).

### Western blot.

Cells were collected and lysed by RIPA buffer with protease inhibitor cocktail (Roche) for 30 minutes on ice. After centrifuging at 12,000*g*, the supernatant was collected and quantified by BCA protein assay kit (Thermo Fisher Scientific). About 30 μg protein was resolved by SDS-PAGE and transferred to PVDF membrane; the membrane was incubated with primary antibodies (flag: MilliporeSigma, F1804; actin: Proteintech, 66009-1-Ig; SLC7A8: Abcam, ab75610) overnight at 4°C and secondary antibodies (anti–mouse IgG, HRP-linked antibody 7076) for 1 hour at room temperature. The target bands were exposed by Amersham Imager 600.

### Statistics.

A nonparametric Kruskal-Wallis rank-sum test was used to compare the statistical difference of multiple independent samples, and Wilcoxon’s rank-sum test was used for intragroup statistical differences. For multiple comparisons, 2-way ANOVA followed by Tukey’s post hoc test was used to analyze the differences in growth and apoptosis rate between experimental groups. *P* values of less than 0.05 were considered significant.

### Study approval.

Study protocols were approved by the Ethics Review Committee of Anhui Provincial Cancer Hospital, Anhui Province, China. All patients provided written informed consent.

## Author contributions

QJM contributed to data curation, methodology, visualization, and writing of the original draft. YW contributed to conceptualization, resources, data curation, investigation, and review and editing of the manuscript. QNW contributed to data curation, validation, and funding acquisition. XFL contributed to data curation and methodology. HJT contributed to methodology and visualization. JWF contributed to data curation and resources. YRC contributed to data curation and visualization. PSF contributed to conceptualization, resources, investigation, and supervision. QMZ contributed to conceptualization, resources, data curation, supervision, funding acquisition, methodology, project administration, and review and editing of the manuscript. The order of coauthorship was based on relevance of contributions to this project.

## Supplementary Material

Supplemental data

Trial reporting checklists

ICMJE disclosure forms

## Version 1 09/08/2021

Electronic publication

## References

[1] Bray F (2018). Global cancer statistics 2018: GLOBOCAN estimates of incidence and mortality worldwide for 36 cancers in 185 countries. CA Cancer J Clin.

[2] Pennathur A (2013). Oesophageal carcinoma. Lancet.

[3] Njei B (2016). Trends in esophageal cancer survival in United States adults from 1973 to 2009: a SEER database analysis. J Gastroenterol Hepatol.

[4] Rustgi AK, El-Serag HB (2014). Esophageal carcinoma. N Engl J Med.

[5] Smyth EC (2017). Oesophageal cancer. Nat Rev Dis Primers.

[6] Coleman HG (2018). The epidemiology of esophageal adenocarcinoma. Gastroenterology.

[7] Napier KJ (2014). Esophageal cancer: a review of epidemiology, pathogenesis, staging workup and treatment modalities. World J Gastrointest Oncol.

[8] Kang X (2015). Personalized targeted therapy for esophageal squamous cell carcinoma. World J Gastroenterol.

[9] Kato H, Nakajima M (2013). Treatments for esophageal cancer: a review. Gen Thorac Cardiovasc Surg.

[10] Park R (2018). Immune therapeutics in the treatment of advanced gastric and esophageal cancer. Anticancer Res.

[11] Liu YB (2017). Evaluation of clinical efficacy after combinational treatment of esophageal cancer using target artery perfusion of verapamil and chemotherapy. Transl Cancer Res.

[12] Mctavish D, Sorkin EM (1989). Verapamil. An updated review of its pharmacodynamic and pharmacokinetic properties, and therapeutic use in hypertension. Drugs.

[13] Grace AA, Camm AJ (2000). Voltage-gated calcium-channels and antiarrhythmic drug action. Cardiovasc Res.

[14] Weiss AT (1983). The use of calcium with verapamil in the management of supraventricular tachyarrhythmias. Int J Cardiol.

[15] Sharom FJ (1997). The P-glycoprotein efflux pump: how does it transport drugs?. J Membr Biol.

[16] Amin ML (2013). P-glycoprotein inhibition for optimal drug delivery. Drug Target Insights.

[17] Zhu T (2017). The effect of verapamil, a P-glycoprotein inhibitor, on the pharmacokinetics of peficitinib, an orally administered, once-daily JAK inhibitor. Clin Pharmacol Drug Dev.

[18] Bansal T (2009). Effect of P-glycoprotein inhibitor, verapamil, on oral bioavailability and pharmacokinetics of irinotecan in rats. Eur J Pharm Sci.

[19] Liu Y (2011). Clinical efficacy of chemotherapy combined with verapamil in metastatic colorectal patients. Cell Biochem Biophys.

[20] Huang J (2013). Clinical evaluation of targeted arterial perfusion of verapamil and chemotherapeutic drugs in interventional therapy of advanced lung cancer. Cancer Chemother Pharmacol.

[21] Pingsheng F (2012). Basic and clinical research on the therapeutic effect of intervention in primary liver cancer by targeted intra-arterial verapamil infusion. Cell Biochem Biophys.

[22] Huang J (2011). Clinical evaluation of targeted arterial infusion of verapamil in the interventional chemotherapy of primary hepatocellular carcinoma. Cell Biochem Biophys.

[23] Ning Z (2014). Efficacy of chemotherapy combined with targeted arterial infusion of verapamil in patients with advanced gastric cancer. Cell Biochem Biophys.

[24] Baguley BC (2010). Multiple drug resistance mechanisms in cancer. Mol Biotechnol.

[25] Housman G (2014). Drug resistance in cancer: an overview. Cancers (Basel).

[26] Kibria G (2014). Cancer multidrug resistance: mechanisms involved and strategies for circumvention using a drug delivery system. Arch Pharm Res.

[27] Song Y (2014). Identification of genomic alterations in oesophageal squamous cell cancer. Nature.

[28] Cheng C (2016). Whole-genome sequencing reveals diverse models of structural variations in esophageal squamous cell carcinoma. Am J Hum Genet.

[29] Tanaka K (2007). Frequent methylation-associated silencing of a candidate tumor-suppressor, CRABP1, in esophageal squamous-cell carcinoma. Oncogene.

[30] Pu W (2017). Targeted bisulfite sequencing identified a panel of DNA methylation-based biomarkers for esophageal squamous cell carcinoma (ESCC). Clin Epigenetics.

[31] Lu T (2019). Identification of DNA methylation-driven genes in esophageal squamous cell carcinoma: a study based on The Cancer Genome Atlas. Cancer Cell Int.

[32] Hao JJ (2016). Spatial intratumoral heterogeneity and temporal clonal evolution in esophageal squamous cell carcinoma. Nat Genet.

[33] Sang MX (2018). Circular RNA ciRS-7 accelerates ESCC progression through acting as a miR-876-5p sponge to enhance MAGE-A family expression. Cancer Lett.

[34] Gao YB (2014). Genetic landscape of esophageal squamous cell carcinoma. Nat Genet.

[35] Lin DC (2014). Genomic and molecular characterization of esophageal squamous cell carcinoma. Nat Genet.

[36] Sawada G (2016). Genomic landscape of esophageal squamous cell carcinoma in a Japanese population. Gastroenterology.

[37] Du P (2017). Comprehensive genomic analysis of oesophageal squamous cell carcinoma reveals clinical relevance. Sci Rep.

[38] Hiyoshi Y (2009). MicroRNA-21 regulates the proliferation and invasion in esophageal squamous cell carcinoma. Clin Cancer Res.

[39] Mori Y (2009). MicroRNA-21 induces cell proliferation and invasion in esophageal squamous cell carcinoma. Mol Med Rep.

[40] Ma WJ (2011). Role of microRNA-21 and effect on PTEN in Kazakh’s esophageal squamous cell carcinoma. Mol Biol Rep.

[41] Harata K (2010). MicroRNA-34b has an oncogenic role in esophageal squamous cell carcinoma. Oncol Lett.

[42] Chen ZL (2011). microRNA-92a promotes lymph node metastasis of human esophageal squamous cell carcinoma via E-cadherin. J Biol Chem.

[43] Chen J (2015). Esophageal squamous cell carcinoma (ESCC): advance in genomics and molecular genetics. Dis Esophagus.

[44] Gisselsson D (2000). Chromosomal breakage-fusion-bridge events cause genetic intratumor heterogeneity. Proc Natl Acad Sci U S A.

[45] Campbell PJ (2010). The patterns and dynamics of genomic instability in metastatic pancreatic cancer. Nature.

[46] Turajlic S (2019). Resolving genetic heterogeneity in cancer. Nat Rev Genet.

[47] Greaves M, Maley CC (2012). Clonal evolution in cancer. Nature.

[48] Ding L (2012). Clonal evolution in relapsed acute myeloid leukaemia revealed by whole-genome sequencing. Nature.

[49] Landau DA (2014). Clonal evolution in hematological malignancies and therapeutic implications. Leukemia.

[50] Turner NC, Reis-Filho JS (2012). Genetic heterogeneity and cancer drug resistance. Lancet Oncol.

[51] Wang H (2014). Methylation of SFRP5 is related to multidrug resistance in leukemia cells. Cancer Gene Ther.

[52] Chen Q (2016). Effectiveness evaluation of organized screening for esophageal cancer: a case-control study in Linzhou city, China. Sci Rep.

[53] Jamal-Hanjani M (2017). Tracking the evolution of non-small-cell lung cancer. N Engl J Med.

[54] Yan T (2019). Multi-region sequencing unveils novel actionable targets and spatial heterogeneity in esophageal squamous cell carcinoma. Nat Commun.

[55] Herling CD (2018). Clonal dynamics towards the development of venetoclax resistance in chronic lymphocytic leukemia. Nat Commun.

[56] Mitchell TJ (2018). Timing the landmark events in the evolution of clear cell renal cell cancer: TRACERx renal. Cell.

[57] Cui Y (2020). Whole-genome sequencing of 508 patients identifies key molecular features associated with poor prognosis in esophageal squamous cell carcinoma. Cell Res.

[58] Stalbovskaya V (2020). NRG1 fusion-driven cancers: a systematic literature review and meta-analysis. J Clin Oncol.

[59] Heining C (2018). *NRG1* fusions in *KRAS* wild-type pancreatic cancer. Cancer Discov.

[60] Wu M (2019). A PLK1 kinase inhibitor enhances the chemosensitivity of cisplatin by inducing pyroptosis in oesophageal squamous cell carcinoma. EBioMedicine.

[61] Teng H (2020). Inter- and intratumor DNA methylation heterogeneity associated with lymph node metastasis and prognosis of esophageal squamous cell carcinoma. Theranostics.

[62] Issa ME (2017). Epigenetic strategies to reverse drug resistance in heterogeneous multiple myeloma. Clin Epigenetics.

[63] Sheffield NC (2017). DNA methylation heterogeneity defines a disease spectrum in Ewing sarcoma. Nat Med.

[64] Zhang L (2015). Genomic analyses reveal mutational signatures and frequently altered genes in esophageal squamous cell carcinoma. Am J Hum Genet.

[65] Dagogo-Jack I, Shaw AT (2018). Tumour heterogeneity and resistance to cancer therapies. Nat Rev Clin Oncol.

[66] Li GM (2008). Mechanisms and functions of DNA mismatch repair. Cell Res.

[67] Aziz MH, Ahmad A (2020). Epigenetic basis of cancer drug resistance. Cancer Drug Resist.

[68] Romero-Garcia S (2020). Role of DNA methylation in the resistance to therapy in solid tumors. Front Oncol.

[69] Saito Y (2019). LLGL2 rescues nutrient stress by promoting leucine uptake in ER^+^ breast cancer. Nature.

[70] Wang Y (2017). GSA: genome sequence archive. Genomics Proteomics Bioinformatics.

[71] Members CN, Partners (2021). Database resources of the National Genomics Data Center, China National Center for Bioinformation in 2021. Nucleic Acids Res.

[72] Martin M (2011). Cutadapt removes adapter sequences from high-throughput sequencing reads. EMBnetjournal.

[73] Li H, Durbin R (2009). Fast and accurate short read alignment with Burrows-Wheeler transform. Bioinformatics.

[74] McKenna A (2010). The Genome Analysis Toolkit: a MapReduce framework for analyzing next-generation DNA sequencing data. Genome Res.

[75] Wang K (2010). ANNOVAR: functional annotation of genetic variants from high-throughput sequencing data. Nucleic Acids Res.

[76] Mayakonda A (2018). Maftools: efficient and comprehensive analysis of somatic variants in cancer. Genome Res.

[77] Boeva V (2012). Control-FREEC: a tool for assessing copy number and allelic content using next-generation sequencing data. Bioinformatics.

[78] Chang J (2017). Genomic analysis of oesophageal squamous-cell carcinoma identifies alcohol drinking-related mutation signature and genomic alterations. Nat Commun.

[79] Cancer Genome Atlas Research Network (2017). Integrated genomic characterization of oesophageal carcinoma. Nature.

[80] Qin H-D (2016). Genomic characterization of esophageal squamous cell carcinoma reveals critical genes underlying tumorigenesis and poor prognosis. Am J Hum Genet.

[81] Lin DC (2018). Genomic and epigenomic aberrations in esophageal squamous cell carcinoma and implications for patients. Gastroenterology.

[82] Lawrence MS (2014). Discovery and saturation analysis of cancer genes across 21 tumour types. Nature.

[83] Tate JG (2019). COSMIC: the catalogue of somatic mutations in cancer. Nucleic Acids Research.

[84] Kumar P (2009). Predicting the effects of coding non-synonymous variants on protein function using the SIFT algorithm. Nat Protoc.

[85] Adzhubei IA (2010). A method and server for predicting damaging missense mutations. Nat Methods.

[86] Schwarz JM (2014). MutationTaster2: mutation prediction for the deep-sequencing age. Nat Methods.

[87] Kasar S (2015). Whole-genome sequencing reveals activation-induced cytidine deaminase signatures during indolent chronic lymphocytic leukaemia evolution. Nat Commun.

[88] Kim J (2016). Somatic ERCC2 mutations are associated with a distinct genomic signature in urothelial tumors. Nat Genet.

[89] Blokzijl F (2018). MutationalPatterns: comprehensive genome-wide analysis of mutational processes. Genome Med.

[90] Alexandrov LB (2020). The repertoire of mutational signatures in human cancer. Nature.

[91] Roth A (2014). PyClone: statistical inference of clonal population structure in cancer. Nat Methods.

[92] Malikic S (2015). Clonality inference in multiple tumor samples using phylogeny. Bioinformatics.

[93] Baum BR (1989). PHYLIP: Phylogeny Inference Package. Version 3.2. Joel Felsenstein. Q Rev Biol.

[94] Guo W (2013). BS-Seeker2: a versatile aligning pipeline for bisulfite sequencing data. BMC Genomics.

[95] Krueger F, Andrews SR (2011). Bismark: a flexible aligner and methylation caller for Bisulfite-Seq applications. Bioinformatics.

[96] Guo WL (2018). CGmapTools improves the precision of heterozygous SNV calls and supports allele-specific methylation detection and visualization in bisulfite-sequencing data. Bioinformatics.

[97] Roadmap Epigenomics C (2015). Integrative analysis of 111 reference human epigenomes. Nature.

[98] Li S (2014). Dynamic evolution of clonal epialleles revealed by methclone. Genome Biol.

[99] Chou TC (2006). Theoretical basis, experimental design, and computerized simulation of synergism and antagonism in drug combination studies. Pharmacol Rev.

