# Supplementary material for: Genomic and epigenomic evolution of acquired resistance to combination therapy in esophageal squamous cell carcinoma
Source: JCI Insight. 2021 Sep 8;6(17):e150203. doi: 10.1172/jci.insight.150203 (PMC8492345; doi:10.1172/jci.insight.150203)
Supplement: Supplemental data [file jciinsight-6-150203-s206.pdf]

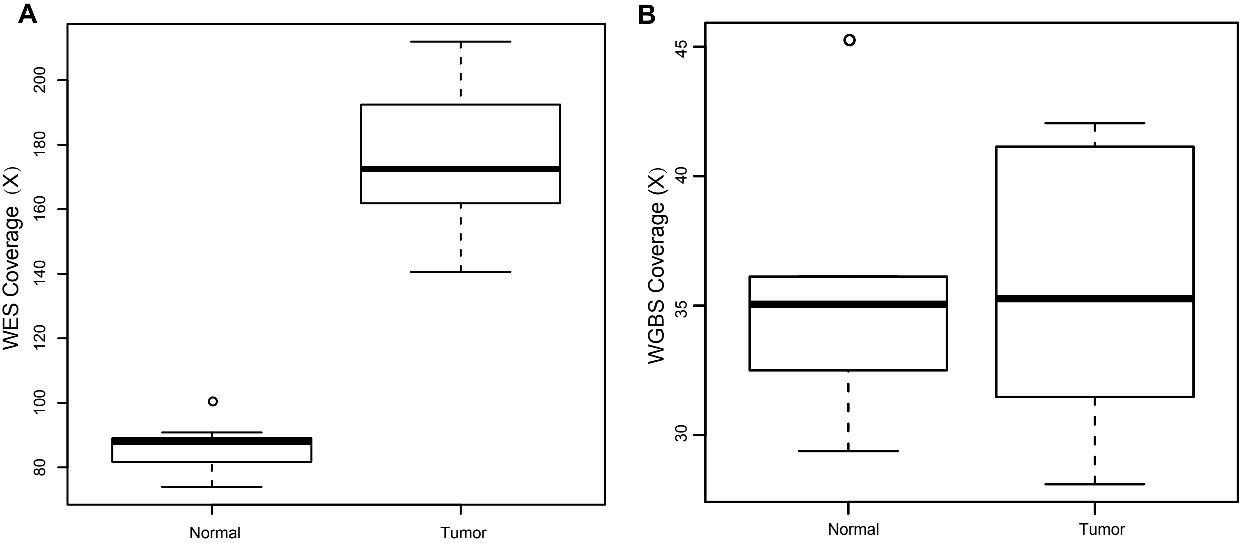

Supplementary Figure 1. Coverage of whole-exome and whole-genome bisulfite sequencing. (**A**) Box plot depicts the coverage of whole-exome sequencing samples. Median coverage was labelled as lines in the central position of box. The first and third quartiles coverages were showed as lines outside of box. Normal (n=12), Tumor (n=16). (**B**) The coverage of whole-genome bisulfite sequencing samples. Normal (n=5), Tumor (n=6).

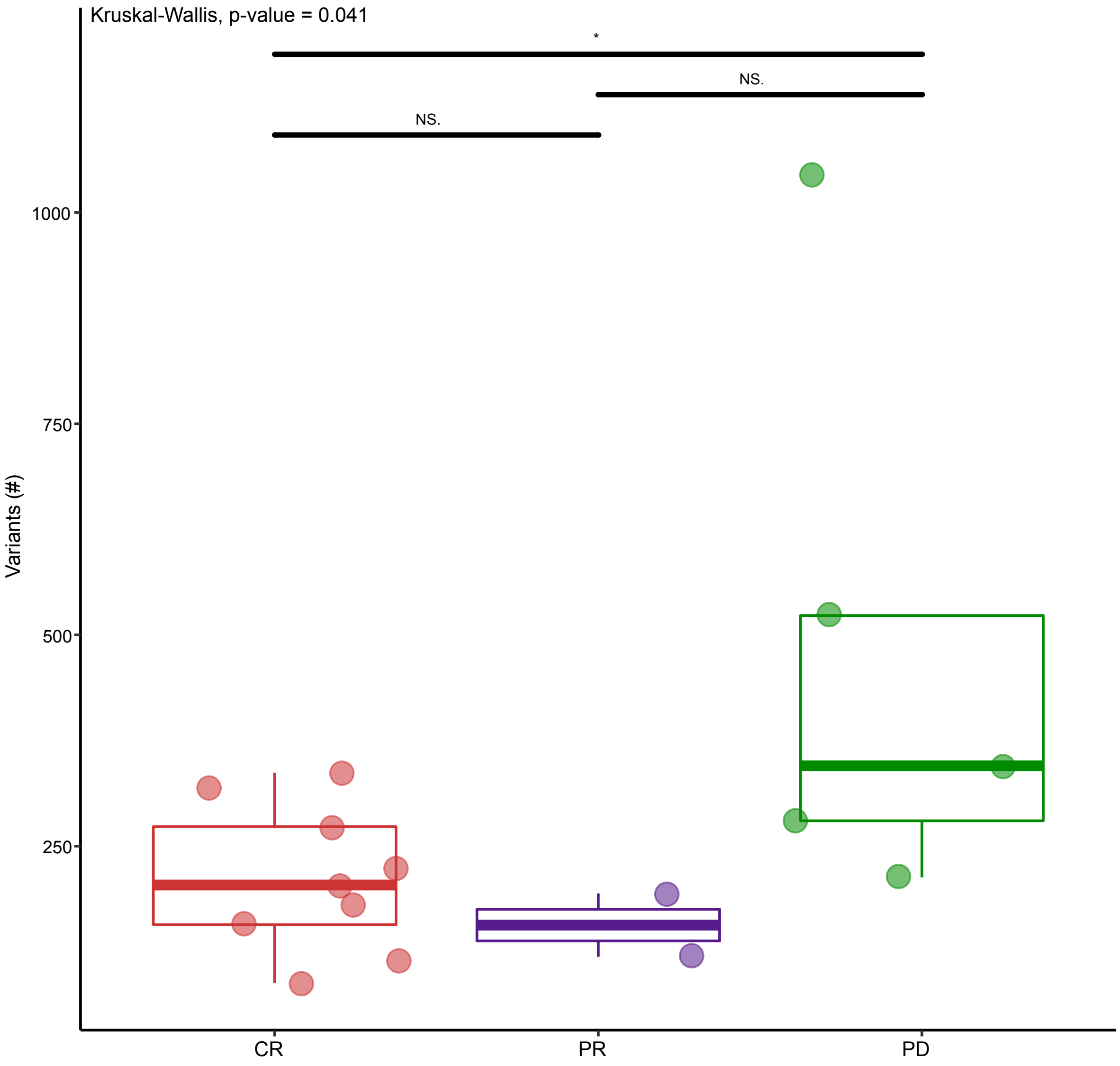

Supplementary Figure 2. Somatic variants in three groups. Boxplot showing somatic variants in different groups. Kruskal-Wallis rank sum test for all groups. Wilcoxon Rank Sum test for each two groups. CR, complete response (n = 9). PR, partial response (n = 2). PD, progressive disease (n = 5).

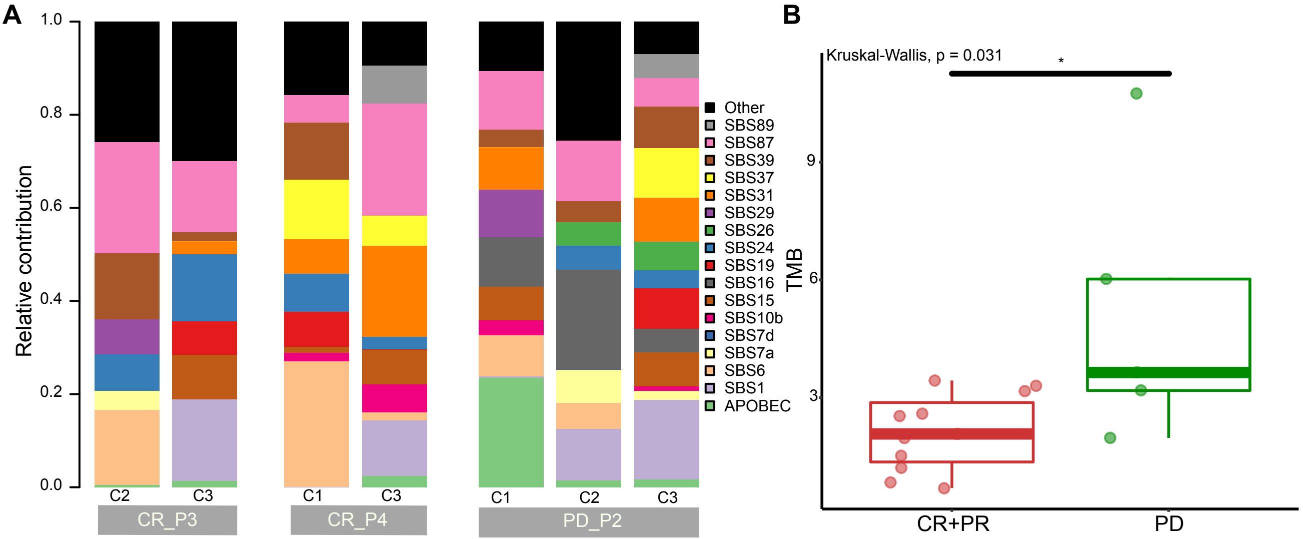

Supplementary Figure 3. Mutation signatures in three patientsand burden. (**A**) Changes of relative mutation signatures contribution between pre-treatment and treatment stages in CR_P3, CR_P4 and PD_P2. (**B**) Boxplot showing mutation burden between PD (n = 5) and non-PD groups (n = 11). CR, complete response. PR, partial response. PD, progressive disease. Kruskal-Wallis rank sum test.

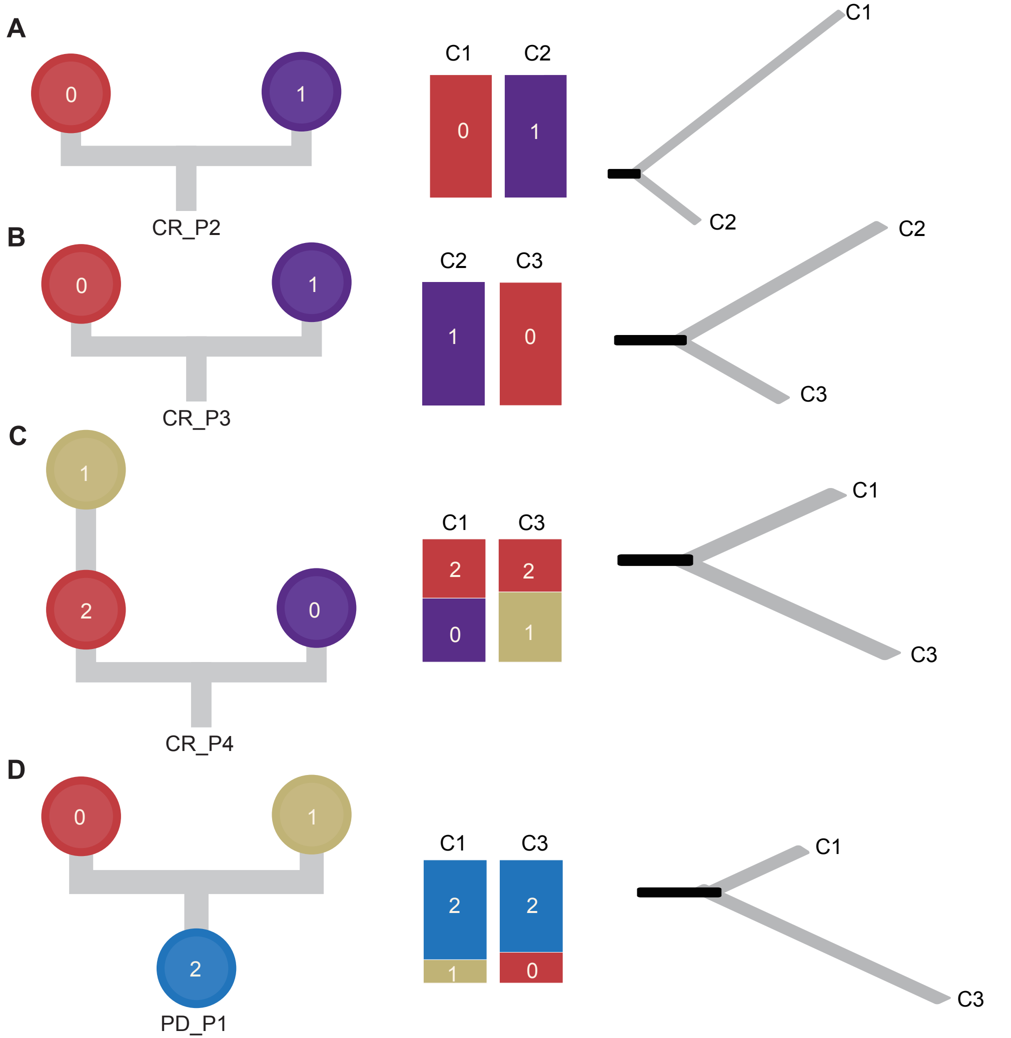

Supplementary Figure 4. Heterogeneity of phylogenetic trees under combined therapies. As shown in Figure 4, the left side of each panel shows the evolution of clone or subclone. The middle of each panel demonstrates the dynamics of clonal composition at each checkpoint across every sample under selective pressures. A discrete-characters parsimony method is used to generate phylogenetic relations based on somatic mutations and copy number aberrations, and showed at the right of each panel. Black: trunk, dark gray: shared branches, gray: private branches. Diverse evolutionary paths were inferred across patients: (**A**) and (**B**) divergent evolution (CR_P2, CR_P3), (**C**) unrooted branching evolution (CR_P4), and (**D**) rooted branching evolution (PD_P1).

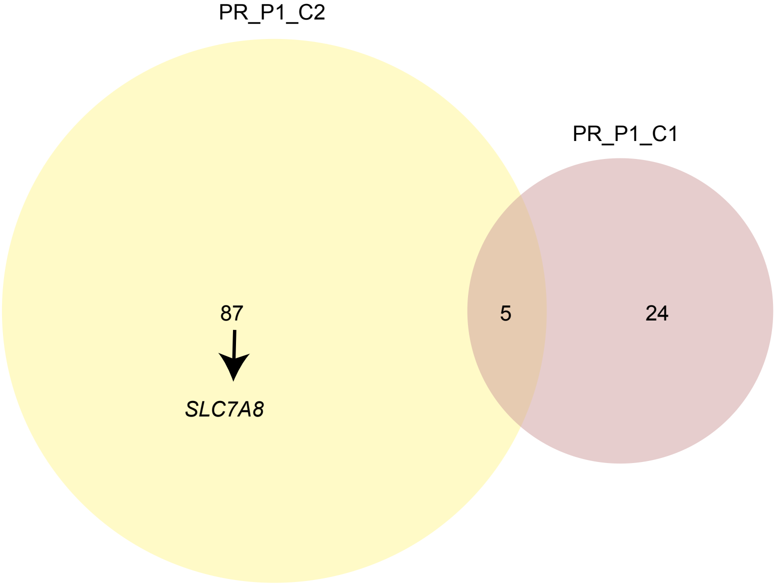

Supplementary Figure 5. Treatment-specific mutation in patient PR_P1. Venn diagram demonstrates the acquired SLC7A8 mutation after combined therapy in PR_P1 patient.
