# Supplementary material for: Genomic and epigenomic evolution of acquired resistance to combination therapy in esophageal squamous cell carcinoma
Source: JCI Insight. 2021 Sep 8;6(17):e150203. doi: 10.1172/jci.insight.150203 (PMC8492345; doi:10.1172/jci.insight.150203)
Supplement: ICMJE disclosure forms [file jciinsight-6-150203-s208.pdf]

ICMJE DISCLOSURE FORM

Date:_ June 12, 2021____
Your Name:_Qingjie Min ____
Manuscript Title:_ Genomic and epigenomic evolution of acquired resistance to chemotherapy combined with verapamil in esophageal squamous cell carcinoma____
Manuscript number (if known):_ 150203-INS-CMED-1____

In the interest of transparency, we ask you to disclose all relationships/activities/interests listed below that are related to the content of your manuscript. “Related” means any relation with for-profit or not-for-profit third parties whose interests may be affected by the content of the manuscript. Disclosure represents a commitment to transparency and does not necessarily indicate a bias. If you are in doubt about whether to list a relationship/activity/interest, it is preferable that you do so.

The following questions apply to the author’s relationships/activities/interests as they relate to the <u>current manuscript only</u>.

The author’s relationships/activities/interests should be <u>defined broadly</u>. For example, if your manuscript pertains to the epidemiology of hypertension, you should declare all relationships with manufacturers of antihypertensive medication, even if that medication is not mentioned in the manuscript.

In item #1 below, report all support for the work reported in this manuscript without time limit. For all other items, the time frame for disclosure is the past 36 months.

		**Name all entities with whom you have this relationship or indicate none (add rows as needed)**	**Specifications/Comments (e.g., if payments were made to you or to your institution)**
		**Time frame: Since the initial planning of the work**	
1	All support for the present manuscript (e.g., funding, provision of study materials, medical writing, article processing charges, etc.) **No time limit for this item.**	National Natural Science Foundation of China	
		Science Foundation of Peking University Cancer Hospital	
		CAMS Innovation Fund for Medical Sciences	
		Major Program of Shenzhen Bay Laboratory	
		Guangdong Basic and Applied Basic Research Foundation	
		The third round of public welfare development and reform pilot projects of Beijing Municipal Medical Research Institutes	

		NATIONAL INSTITUTE OF HEALTH DATA SCIENCE OF PEKING UNIVERSITY	
**Time frame: past 36 months**			
2	Grants or contracts from any entity (if not indicated in item #1 above).	____ None	
3	Royalties or licenses	____ None	
4	Consulting fees	____ None	
5	Payment or honoraria for lectures, presentations, speakers bureaus, manuscript writing or educational events	____ None	
6	Payment for expert testimony	____ None	
7	Support for attending meetings and/or travel	____ None	
8	Patents planned, issued or pending	____ None	
9	Participation on a Data Safety Monitoring Board or Advisory Board	____ None	
10	Leadership or fiduciary role in other board, society, committee or advocacy group, paid or unpaid	____ None	
11	Stock or stock options	____ None	
12	Receipt of equipment, materials, drugs, medical writing, gifts or other services	____ None	

13	Other financial or non-financial interests	____ None	

Please place an "X" next to the following statement to indicate your agreement:

_ X __ I certify that I have answered every question and have not altered the wording of any of the questions on this form.

ICMJE DISCLOSURE FORM

Date:_ June 12, 2021____
Your Name:_Yan Wang ____
Manuscript Title:_ Genomic and epigenomic evolution of acquired resistance to chemotherapy combined with verapamil in esophageal squamous cell carcinoma____
Manuscript number (if known):_ 150203-INS-CMED-1____

In the interest of transparency, we ask you to disclose all relationships/activities/interests listed below that are related to the content of your manuscript. "Related" means any relation with for-profit or not-for-profit third parties whose interests may be affected by the content of the manuscript. Disclosure represents a commitment to transparency and does not necessarily indicate a bias. If you are in doubt about whether to list a relationship/activity/interest, it is preferable that you do so.

The following questions apply to the author's relationships/activities/interests as they relate to the <u>current manuscript only</u>.

The author's relationships/activities/interests should be <u>defined broadly</u>. For example, if your manuscript pertains to the epidemiology of hypertension, you should declare all relationships with manufacturers of antihypertensive medication, even if that medication is not mentioned in the manuscript.

In item #1 below, report all support for the work reported in this manuscript without time limit. For all other items, the time frame for disclosure is the past 36 months.

		**Name all entities with whom you have this relationship or indicate none (add rows as needed)**	**Specifications/Comments (e.g., if payments were made to you or to your institution)**
		**Time frame: Since the initial planning of the work**	
1	All support for the present manuscript (e.g., funding, provision of study materials, medical writing, article processing charges, etc.) **No time limit for this item.**	National Natural Science Foundation of China	
		Science Foundation of Peking University Cancer Hospital	
		CAMS Innovation Fund for Medical Sciences	
		Major Program of Shenzhen Bay Laboratory	
		Guangdong Basic and Applied Basic Research Foundation	
		The third round of public welfare development and reform pilot projects of Beijing Municipal Medical Research Institutes	

		NATIONAL INSTITUTE OF HEALTH DATA SCIENCE OF PEKING UNIVERSITY	
**Time frame: past 36 months**			
2	Grants or contracts from any entity (if not indicated in item #1 above).	____ None	
3	Royalties or licenses	____ None	
4	Consulting fees	____ None	
5	Payment or honoraria for lectures, presentations, speakers bureaus, manuscript writing or educational events	____ None	
6	Payment for expert testimony	____ None	
7	Support for attending meetings and/or travel	____ None	
8	Patents planned, issued or pending	____ None	
9	Participation on a Data Safety Monitoring Board or Advisory Board	____ None	
10	Leadership or fiduciary role in other board, society, committee or advocacy group, paid or unpaid	____ None	
11	Stock or stock options	____ None	
12	Receipt of equipment, materials, drugs, medical writing, gifts or other services	____ None	

13	Other financial or non-financial interests	____ None	

Please place an "X" next to the following statement to indicate your agreement:

_ X __ I certify that I have answered every question and have not altered the wording of any of the questions on this form.

ICMJE DISCLOSURE FORM

Date:_ June 12, 2021____
Your Name:_Qingnan Wu ____
Manuscript Title:_ Genomic and epigenomic evolution of acquired resistance to chemotherapy combined with verapamil in esophageal squamous cell carcinoma____
Manuscript number (if known):_ 150203-INS-CMED-1____

In the interest of transparency, we ask you to disclose all relationships/activities/interests listed below that are related to the content of your manuscript. "Related" means any relation with for-profit or not-for-profit third parties whose interests may be affected by the content of the manuscript. Disclosure represents a commitment to transparency and does not necessarily indicate a bias. If you are in doubt about whether to list a relationship/activity/interest, it is preferable that you do so.

The following questions apply to the author's relationships/activities/interests as they relate to the <u>current manuscript only</u>.

The author's relationships/activities/interests should be <u>defined broadly</u>. For example, if your manuscript pertains to the epidemiology of hypertension, you should declare all relationships with manufacturers of antihypertensive medication, even if that medication is not mentioned in the manuscript.

In item #1 below, report all support for the work reported in this manuscript without time limit. For all other items, the time frame for disclosure is the past 36 months.

		**Name all entities with whom you have this relationship or indicate none (add rows as needed)**	**Specifications/Comments (e.g., if payments were made to you or to your institution)**
		**Time frame: Since the initial planning of the work**	
1	All support for the present manuscript (e.g., funding, provision of study materials, medical writing, article processing charges, etc.) **No time limit for this item.**	National Natural Science Foundation of China	
		Science Foundation of Peking University Cancer Hospital	
		CAMS Innovation Fund for Medical Sciences	
		Major Program of Shenzhen Bay Laboratory	
		Guangdong Basic and Applied Basic Research Foundation	
		The third round of public welfare development and reform pilot projects of Beijing Municipal Medical Research Institutes	

		NATIONAL INSTITUTE OF HEALTH DATA SCIENCE OF PEKING UNIVERSITY	
**Time frame: past 36 months**			
2	Grants or contracts from any entity (if not indicated in item #1 above).	____ None	
3	Royalties or licenses	____ None	
4	Consulting fees	____ None	
5	Payment or honoraria for lectures, presentations, speakers bureaus, manuscript writing or educational events	____ None	
6	Payment for expert testimony	____ None	
7	Support for attending meetings and/or travel	____ None	
8	Patents planned, issued or pending	____ None	
9	Participation on a Data Safety Monitoring Board or Advisory Board	____ None	
10	Leadership or fiduciary role in other board, society, committee or advocacy group, paid or unpaid	____ None	
11	Stock or stock options	____ None	
12	Receipt of equipment, materials, drugs, medical writing, gifts or other services	____ None	

13	Other financial or non-financial interests	____ None	

Please place an "X" next to the following statement to indicate your agreement:

_ X __ I certify that I have answered every question and have not altered the wording of any of the questions on this form.

ICMJE DISCLOSURE FORM

Date:_ June 12, 2021____
Your Name:_Xianfeng Li ____
Manuscript Title:_ Genomic and epigenomic evolution of acquired resistance to chemotherapy combined with verapamil in esophageal squamous cell carcinoma____
Manuscript number (if known):_ 150203-INS-CMED-1____

In the interest of transparency, we ask you to disclose all relationships/activities/interests listed below that are related to the content of your manuscript. "Related" means any relation with for-profit or not-for-profit third parties whose interests may be affected by the content of the manuscript. Disclosure represents a commitment to transparency and does not necessarily indicate a bias. If you are in doubt about whether to list a relationship/activity/interest, it is preferable that you do so.

The following questions apply to the author's relationships/activities/interests as they relate to the <u>current manuscript only</u>.

The author's relationships/activities/interests should be <u>defined broadly</u>. For example, if your manuscript pertains to the epidemiology of hypertension, you should declare all relationships with manufacturers of antihypertensive medication, even if that medication is not mentioned in the manuscript.

In item #1 below, report all support for the work reported in this manuscript without time limit. For all other items, the time frame for disclosure is the past 36 months.

		**Name all entities with whom you have this relationship or indicate none (add rows as needed)**	**Specifications/Comments (e.g., if payments were made to you or to your institution)**
		**Time frame: Since the initial planning of the work**	
1	All support for the present manuscript (e.g., funding, provision of study materials, medical writing, article processing charges, etc.) **No time limit for this item.**	National Natural Science Foundation of China	
		Science Foundation of Peking University Cancer Hospital	
		CAMS Innovation Fund for Medical Sciences	
		Major Program of Shenzhen Bay Laboratory	
		Guangdong Basic and Applied Basic Research Foundation	
		The third round of public welfare development and reform pilot projects of Beijing Municipal Medical Research Institutes	

		NATIONAL INSTITUTE OF HEALTH DATA SCIENCE OF PEKING UNIVERSITY	
**Time frame: past 36 months**			
2	Grants or contracts from any entity (if not indicated in item #1 above).	____ None	
3	Royalties or licenses	____ None	
4	Consulting fees	____ None	
5	Payment or honoraria for lectures, presentations, speakers bureaus, manuscript writing or educational events	____ None	
6	Payment for expert testimony	____ None	
7	Support for attending meetings and/or travel	____ None	
8	Patents planned, issued or pending	____ None	
9	Participation on a Data Safety Monitoring Board or Advisory Board	____ None	
10	Leadership or fiduciary role in other board, society, committee or advocacy group, paid or unpaid	____ None	
11	Stock or stock options	____ None	
12	Receipt of equipment, materials, drugs, medical writing, gifts or other services	____ None	

13	Other financial or non-financial interests	____ None	

Please place an "X" next to the following statement to indicate your agreement:

_ X __ I certify that I have answered every question and have not altered the wording of any of the questions on this form.

ICMJE DISCLOSURE FORM

Date:_ June 12, 2021____
Your Name:_Huajing Teng ____
Manuscript Title:_ Genomic and epigenomic evolution of acquired resistance to chemotherapy combined with verapamil in esophageal squamous cell carcinoma____
Manuscript number (if known):_ 150203-INS-CMED-1____

In the interest of transparency, we ask you to disclose all relationships/activities/interests listed below that are related to the content of your manuscript. "Related" means any relation with for-profit or not-for-profit third parties whose interests may be affected by the content of the manuscript. Disclosure represents a commitment to transparency and does not necessarily indicate a bias. If you are in doubt about whether to list a relationship/activity/interest, it is preferable that you do so.

The following questions apply to the author's relationships/activities/interests as they relate to the <u>current manuscript only</u>.

The author's relationships/activities/interests should be <u>defined broadly</u>. For example, if your manuscript pertains to the epidemiology of hypertension, you should declare all relationships with manufacturers of antihypertensive medication, even if that medication is not mentioned in the manuscript.

In item #1 below, report all support for the work reported in this manuscript without time limit. For all other items, the time frame for disclosure is the past 36 months.

		**Name all entities with whom you have this relationship or indicate none (add rows as needed)**	**Specifications/Comments (e.g., if payments were made to you or to your institution)**
		**Time frame: Since the initial planning of the work**	
1	All support for the present manuscript (e.g., funding, provision of study materials, medical writing, article processing charges, etc.) **No time limit for this item.**	National Natural Science Foundation of China	
		Science Foundation of Peking University Cancer Hospital	
		CAMS Innovation Fund for Medical Sciences	
		Major Program of Shenzhen Bay Laboratory	
		Guangdong Basic and Applied Basic Research Foundation	
		The third round of public welfare development and reform pilot projects of Beijing Municipal Medical Research Institutes	

		NATIONAL INSTITUTE OF HEALTH DATA SCIENCE OF PEKING UNIVERSITY	
**Time frame: past 36 months**			
2	Grants or contracts from any entity (if not indicated in item #1 above).	____ None	
3	Royalties or licenses	____ None	
4	Consulting fees	____ None	
5	Payment or honoraria for lectures, presentations, speakers bureaus, manuscript writing or educational events	____ None	
6	Payment for expert testimony	____ None	
7	Support for attending meetings and/or travel	____ None	
8	Patents planned, issued or pending	____ None	
9	Participation on a Data Safety Monitoring Board or Advisory Board	____ None	
10	Leadership or fiduciary role in other board, society, committee or advocacy group, paid or unpaid	____ None	
11	Stock or stock options	____ None	
12	Receipt of equipment, materials, drugs, medical writing, gifts or other services	____ None	

13	Other financial or non-financial interests	____ None	

Please place an "X" next to the following statement to indicate your agreement:

_ X __ I certify that I have answered every question and have not altered the wording of any of the questions on this form.

ICMJE DISCLOSURE FORM

Date:_ June 12, 2021____
Your Name:_Jiawen Fan____
Manuscript Title:_ Genomic and epigenomic evolution of acquired resistance to chemotherapy combined with verapamil in esophageal squamous cell carcinoma____
Manuscript number (if known):_ 150203-INS-CMED-1____

In the interest of transparency, we ask you to disclose all relationships/activities/interests listed below that are related to the content of your manuscript. "Related" means any relation with for-profit or not-for-profit third parties whose interests may be affected by the content of the manuscript. Disclosure represents a commitment to transparency and does not necessarily indicate a bias. If you are in doubt about whether to list a relationship/activity/interest, it is preferable that you do so.

The following questions apply to the author's relationships/activities/interests as they relate to the <u>current manuscript only</u>.

The author's relationships/activities/interests should be <u>defined broadly</u>. For example, if your manuscript pertains to the epidemiology of hypertension, you should declare all relationships with manufacturers of antihypertensive medication, even if that medication is not mentioned in the manuscript.

In item #1 below, report all support for the work reported in this manuscript without time limit. For all other items, the time frame for disclosure is the past 36 months.

		**Name all entities with whom you have this relationship or indicate none (add rows as needed)**	**Specifications/Comments (e.g., if payments were made to you or to your institution)**
		**Time frame: Since the initial planning of the work**	
1	All support for the present manuscript (e.g., funding, provision of study materials, medical writing, article processing charges, etc.) **No time limit for this item.**	National Natural Science Foundation of China	
		Science Foundation of Peking University Cancer Hospital	
		CAMS Innovation Fund for Medical Sciences	
		Major Program of Shenzhen Bay Laboratory	
		Guangdong Basic and Applied Basic Research Foundation	
		The third round of public welfare development and reform pilot projects of Beijing Municipal Medical Research Institutes	

		NATIONAL INSTITUTE OF HEALTH DATA SCIENCE OF PEKING UNIVERSITY	
**Time frame: past 36 months**			
2	Grants or contracts from any entity (if not indicated in item #1 above).	____ None	
3	Royalties or licenses	____ None	
4	Consulting fees	____ None	
5	Payment or honoraria for lectures, presentations, speakers bureaus, manuscript writing or educational events	____ None	
6	Payment for expert testimony	____ None	
7	Support for attending meetings and/or travel	____ None	
8	Patents planned, issued or pending	____ None	
9	Participation on a Data Safety Monitoring Board or Advisory Board	____ None	
10	Leadership or fiduciary role in other board, society, committee or advocacy group, paid or unpaid	____ None	
11	Stock or stock options	____ None	
12	Receipt of equipment, materials, drugs, medical writing, gifts or other services	____ None	

13	Other financial or non-financial interests	____ None	

Please place an "X" next to the following statement to indicate your agreement:

_ X __ I certify that I have answered every question and have not altered the wording of any of the questions on this form.

ICMJE DISCLOSURE FORM

Date:_ June 12, 2021____
Your Name:_Yiren Cao____
Manuscript Title:_ Genomic and epigenomic evolution of acquired resistance to chemotherapy combined with verapamil in esophageal squamous cell carcinoma____
Manuscript number (if known):_ 150203-INS-CMED-1____

In the interest of transparency, we ask you to disclose all relationships/activities/interests listed below that are related to the content of your manuscript. "Related" means any relation with for-profit or not-for-profit third parties whose interests may be affected by the content of the manuscript. Disclosure represents a commitment to transparency and does not necessarily indicate a bias. If you are in doubt about whether to list a relationship/activity/interest, it is preferable that you do so.

The following questions apply to the author's relationships/activities/interests as they relate to the <u>current manuscript only</u>.

The author's relationships/activities/interests should be <u>defined broadly</u>. For example, if your manuscript pertains to the epidemiology of hypertension, you should declare all relationships with manufacturers of antihypertensive medication, even if that medication is not mentioned in the manuscript.

In item #1 below, report all support for the work reported in this manuscript without time limit. For all other items, the time frame for disclosure is the past 36 months.

		**Name all entities with whom you have this relationship or indicate none (add rows as needed)**	**Specifications/Comments (e.g., if payments were made to you or to your institution)**
		**Time frame: Since the initial planning of the work**	
1	All support for the present manuscript (e.g., funding, provision of study materials, medical writing, article processing charges, etc.) **No time limit for this item.**	National Natural Science Foundation of China	
		Science Foundation of Peking University Cancer Hospital	
		CAMS Innovation Fund for Medical Sciences	
		Major Program of Shenzhen Bay Laboratory	
		Guangdong Basic and Applied Basic Research Foundation	
		The third round of public welfare development and reform pilot projects of Beijing Municipal Medical Research Institutes	

		NATIONAL INSTITUTE OF HEALTH DATA SCIENCE OF PEKING UNIVERSITY	
**Time frame: past 36 months**			
2	Grants or contracts from any entity (if not indicated in item #1 above).	____ None	
3	Royalties or licenses	____ None	
4	Consulting fees	____ None	
5	Payment or honoraria for lectures, presentations, speakers bureaus, manuscript writing or educational events	____ None	
6	Payment for expert testimony	____ None	
7	Support for attending meetings and/or travel	____ None	
8	Patents planned, issued or pending	____ None	
9	Participation on a Data Safety Monitoring Board or Advisory Board	____ None	
10	Leadership or fiduciary role in other board, society, committee or advocacy group, paid or unpaid	____ None	
11	Stock or stock options	____ None	
12	Receipt of equipment, materials, drugs, medical writing, gifts or other services	____ None	

13	Other financial or non-financial interests	____ None	

Please place an "X" next to the following statement to indicate your agreement:

_ X __ I certify that I have answered every question and have not altered the wording of any of the questions on this form.

ICMJE DISCLOSURE FORM

Date:_ June 12, 2021____
Your Name:_ Pingsheng Fan ____
Manuscript Title:_ Genomic and epigenomic evolution of acquired resistance to chemotherapy combined with verapamil in esophageal squamous cell carcinoma____
Manuscript number (if known):_ 150203-INS-CMED-1____

In the interest of transparency, we ask you to disclose all relationships/activities/interests listed below that are related to the content of your manuscript. "Related" means any relation with for-profit or not-for-profit third parties whose interests may be affected by the content of the manuscript. Disclosure represents a commitment to transparency and does not necessarily indicate a bias. If you are in doubt about whether to list a relationship/activity/interest, it is preferable that you do so.

The following questions apply to the author's relationships/activities/interests as they relate to the <u>current manuscript only</u>.

The author's relationships/activities/interests should be <u>defined broadly</u>. For example, if your manuscript pertains to the epidemiology of hypertension, you should declare all relationships with manufacturers of antihypertensive medication, even if that medication is not mentioned in the manuscript.

In item #1 below, report all support for the work reported in this manuscript without time limit. For all other items, the time frame for disclosure is the past 36 months.

		**Name all entities with whom you have this relationship or indicate none (add rows as needed)**	**Specifications/Comments (e.g., if payments were made to you or to your institution)**
		**Time frame: Since the initial planning of the work**	
1	All support for the present manuscript (e.g., funding, provision of study materials, medical writing, article processing charges, etc.) **No time limit for this item.**	National Natural Science Foundation of China	
		Science Foundation of Peking University Cancer Hospital	
		CAMS Innovation Fund for Medical Sciences	
		Major Program of Shenzhen Bay Laboratory	
		Guangdong Basic and Applied Basic Research Foundation	
		The third round of public welfare development and reform pilot projects of Beijing Municipal Medical Research Institutes	

		NATIONAL INSTITUTE OF HEALTH DATA SCIENCE OF PEKING UNIVERSITY	
**Time frame: past 36 months**			
2	Grants or contracts from any entity (if not indicated in item #1 above).	____ None	
3	Royalties or licenses	____ None	
4	Consulting fees	____ None	
5	Payment or honoraria for lectures, presentations, speakers bureaus, manuscript writing or educational events	____ None	
6	Payment for expert testimony	____ None	
7	Support for attending meetings and/or travel	____ None	
8	Patents planned, issued or pending	____ None	
9	Participation on a Data Safety Monitoring Board or Advisory Board	____ None	
10	Leadership or fiduciary role in other board, society, committee or advocacy group, paid or unpaid	____ None	
11	Stock or stock options	____ None	
12	Receipt of equipment, materials, drugs, medical writing, gifts or other services	____ None	

13	Other financial or non-financial interests	____ None	

Please place an "X" next to the following statement to indicate your agreement:

_ X __ I certify that I have answered every question and have not altered the wording of any of the questions on this form.

ICMJE DISCLOSURE FORM

Date:_ June 12, 2021____
Your Name:_Qimin Zhan ____
Manuscript Title:_ Genomic and epigenomic evolution of acquired resistance to chemotherapy combined with verapamil in esophageal squamous cell carcinoma____
Manuscript number (if known):_ 150203-INS-CMED-1____

In the interest of transparency, we ask you to disclose all relationships/activities/interests listed below that are related to the content of your manuscript. "Related" means any relation with for-profit or not-for-profit third parties whose interests may be affected by the content of the manuscript. Disclosure represents a commitment to transparency and does not necessarily indicate a bias. If you are in doubt about whether to list a relationship/activity/interest, it is preferable that you do so.

The following questions apply to the author's relationships/activities/interests as they relate to the current manuscript only.

The author's relationships/activities/interests should be defined broadly. For example, if your manuscript pertains to the epidemiology of hypertension, you should declare all relationships with manufacturers of antihypertensive medication, even if that medication is not mentioned in the manuscript.

In item #1 below, report all support for the work reported in this manuscript without time limit. For all other items, the time frame for disclosure is the past 36 months.

		**Name all entities with whom you have this relationship or indicate none (add rows as needed)**	**Specifications/Comments (e.g., if payments were made to you or to your institution)**
		**Time frame: Since the initial planning of the work**	
1	All support for the present manuscript (e.g., funding, provision of study materials, medical writing, article processing charges, etc.) **No time limit for this item.**	National Natural Science Foundation of China	
		Science Foundation of Peking University Cancer Hospital	
		CAMS Innovation Fund for Medical Sciences	
		Major Program of Shenzhen Bay Laboratory	
		Guangdong Basic and Applied Basic Research Foundation	
		The third round of public welfare development and reform pilot projects of Beijing Municipal Medical Research Institutes	

		NATIONAL INSTITUTE OF HEALTH DATA SCIENCE OF PEKING UNIVERSITY	
**Time frame: past 36 months**			
2	Grants or contracts from any entity (if not indicated in item #1 above).	____ None	
3	Royalties or licenses	____ None	
4	Consulting fees	____ None	
5	Payment or honoraria for lectures, presentations, speakers bureaus, manuscript writing or educational events	____ None	
6	Payment for expert testimony	____ None	
7	Support for attending meetings and/or travel	____ None	
8	Patents planned, issued or pending	____ None	
9	Participation on a Data Safety Monitoring Board or Advisory Board	____ None	
10	Leadership or fiduciary role in other board, society, committee or advocacy group, paid or unpaid	____ None	
11	Stock or stock options	____ None	
12	Receipt of equipment, materials, drugs, medical writing, gifts or other services	____ None	

13	Other financial or non-financial interests	____ None	

Please place an "X" next to the following statement to indicate your agreement:

_ X __ I certify that I have answered every question and have not altered the wording of any of the questions on this form.
